# Neutral molecular markers support common origin of aluminium tolerance in three congeneric grass species growing in acidic soils

**DOI:** 10.1093/aobpla/plx060

**Published:** 2017-11-07

**Authors:** Roberto Contreras, Ana M Figueiras, F Javier Gallego, Elena Benavente, Antonio J Manzaneda, César Benito

**Affiliations:** Departamento de Genética, Facultad de Biología, Universidad Complutense de Madrid, Madrid, Spain; Departamento de Biotecnología-Biología Vegetal, Escuela Técnica Superior de Ingenieros Agrónomos, Universidad Politécnica de Madrid, Madrid, Spain; Departamento de Biología Animal, Biología Vegetal y Ecología, Universidad de Jaén, Paraje Las Lagunillas s⁄n, Jaén, Spain

**Keywords:** Acidic soils, aluminium, *Brachypodium distachyon*, *Brachypodium hybridum*, tolerance

## Abstract

Aluminium (Al) toxicity is the main abiotic stress limiting plant productivity in acidic soils that are widely distributed among arable lands. Plant species differ in the level of Al resistance showing intraspecific and interspecific variation in many crop species. However, the origin of Al-tolerance is not well known. Three annual species, difficult to distinguish phenotypically and that were until recently misinterpreted as a single complex species under *Brachypodium distachyon*, have been recently separated into three distinct species: the diploids *B. distachyon* (2*n* = 10) and *B. stacei* (2*n* = 20), and *B. hybridum* (2*n* = 30), the allotetraploid derived from the two diploid species. The aims of this work were to know the origin of Al-tolerance in acidic soil conditions within these three *Brachypodium* species and to develop new DNA markers for species discrimination. Two multiplex SSR-PCRs allowed to genotype a group of 94 accessions for 17 pentanucleotide microsatellite (SSRs) loci. The variability for 139 inter-microsatellite (ISSRs) markers was also examined. The genetic relationships obtained using those neutral molecular markers (SSRs and ISSRs) support that all Al-tolerant allotetraploid accessions of *B. hybridum* have a common origin that is related with both geographic location and acidic soils. The possibility that the adaptation to acidic soils caused the isolation of the tolerant *B. hybridum* populations from the others is discussed. We finally describe a new, easy, DNA barcoding method based in the upstream-intron 1 region of the *ALMT1* gene, a tool that is 100 % effective to distinguish among these three *Brachypodium* species.

## Teaser

Aluminium (Al) toxicity is the main abiotic stress limiting plant productivity in acidic soils that are widely distributed among arable lands. Plant species differ in the level of Al resistance showing intraspecific and interspecific variation in many crop species. However, the origin of Al tolerance is not well known. Our most important result is that all Al-tolerant B. hybridum allotetraploid accessions from the western areas of the Iberian Peninsula (areas with acidic soils) have a common origin. Moreover, we have developed several new molecular markers that discriminate very easily among B. distachyon, B. stacei and B. hybridum.

## Introduction

Aluminium (Al) is the most abundant metal in the crust of the Earth. This metal is toxic on acidic soils and severely limits plant growth. Under acidic environment (pH < 5) the rhizotoxic Al^3+^ is solubilized and the root growth is inhibited (Kochian *et al.* 2004, 2005). Approximately 30 % of the Earth’s total land area consists of highly acidic soils, and as much as 50 % of the world’s potentially arable lands are acidic (von Uexküll and Mutert 1995). The tropic and subtropics are important food-producing regions. These regions comprise large areas of acidic soils that are also very frequent in the Iberian Peninsula. The main abiotic stress limiting crop production is the drought and the second is Al toxicity both constituting food security threats (von Uexküll and Mutert 1995). The study of complex stress tolerance traits like Al-tolerance is difficult in important agronomical species like wheat, barley and rye, which have large genomes, abundant repetitive sequences and some, as wheat, are polyploids. Previous studies, using candidate Al-tolerance genes, have suggested that several tolerant varieties of wheat and barley were originated from acidic soils (Ryan *et al.* 2010; Ryan and Delhaize 2010; Delhaize *et al.* 2012a; Fujii *et al.* 2012; Garcia-Oliveira *et al.* 2013, 2014). Al-tolerance is common in species endemic to regions with acidic soils where the ability to cope with Al^3+^ stress is a prerequisite for survival. Examples include tea (*Camellia sinensis*), buckwheat (*Fagopyrum esculentum*), *Melostoma* and *Hydrangea* sp., all of which accumulate high concentrations of Al in their leaves (Ma *et al.* 2001; Ryan *et al.* 2010). However, there are no genetic studies with neutral molecular markers indicating a common origin for Al-tolerance in acidic soils. The origins of Al resistance in wheat (*Triticum aestivum*) are difficult to explain because all the diploid progenitors of this hexaploid species are reportedly sensitive to Al stress. The expression of an anion channel (TaALMT1) that releases malate anions from the root apices controls the genotypic variation for Al resistance in wheat (Ryan *et al.* 2010). This gene (*TaALMT1*) is not a neutral marker. This trait in wheat has multiple independent origins that enhance Al resistance by increasing *TaALMT1* expression and is an example of evolutionary adaptation to a major abiotic stress (Ryan *et al.* 2010).

*Brachypodium distachyon* is a diploid annual small grass with several attributes suitable for being an excellent genetic model organism for the Poaceae. For example, its morphological characteristics, like a small stature, and simple growth conditions, allow to grow large amounts of plants in a small space in growth chambers and, also, to obtain several generations in a year, with a minimum of 6 weeks from seed to seed (Garvin *et al.* 2008; Mur *et al.* 2011). In addition, as *B. distachyon* is self-fertile (autogamous) plant, it is easy and quick to obtain uniform pure inbred lines composed by plants with identical genotype within two generations (Vogel *et al.* 2009). Moreover, its small (1C = 0.3 pg of DNA), recently sequenced, genome contains mostly single or low-copy repetitive DNA (Wolny and Hasterok 2009; The International *Brachypodium* Initiative [IBI] 2010). On the other hand, it is feasible to obtain transgenic plants in *B. distachyon* due to the existence of efficient Agrobacterium-mediated transformation protocols (Vogel *et al.* 2009). Finally, the phylogenetic studies indicate that *B. distachyon* is genetically closer to economically important Triticeae species like wheat (*Triticum* spp.) and barley (*Hordeum* spp.) than rice (*Oryza sativa*), a subtropical cereal with a fully sequenced genome (Grass Phylogeny Working Group 2001; Catalán *et al.* 2012). Because of all these attributes, *B. distachyon* is a truly tractable model grass for monocot and especially for Triticeae species.

The characterization of natural diversity and the phylogenetic relationships of *B. distachyon*, *B. stacei* and *B. hybridum* species have been carried out using molecular, cytological and morphological markers (Filiz *et al.* 2009; Vogel *et al.* 2009; Mur *et al.* 2011; Catalán *et al.* 2012; López-Álvarez *et al.* 2012; Manzaneda *et al.* 2012). The first cytogenetic analyses of *B. distachyon sensu lato* pointed out the existence of three different putative ploidy levels with 2*n* = 10 (initial diploid race), 2*n* = 20 (autotetraploid) and 2*n* = 30 (autohexaploid) chromosomes (Robertson 1981). However, recent cytogenetic studies using fluorescence *in situ* hybridization with both genomic DNA and ribosomal DNA genes and also using ‘single-locus’ BAC (bacterial artificial chromosome) clones indicated that 2*n* = 10 and 2*n* = 20 chromosome cytotypes were two distinct diploid taxa with a different basic chromosome number of *x* = 5 and *x* = 10, respectively. The 2*n* = 30 chromosome cytotype was the allotetraploid between these two diploid taxa (Hasterok *et al.* 2004, 2006). This finding has also been supported using comparative chromosome painting (Idziak *et al.* 2011). Phylogenetic analysis using five nuclear loci and two plastid sequences demonstrated that the 2*n* = 2*x* = 10 and 2*n* = 2*x* = 20 cytotypes were two independent lineages that were, respectively, the paternal and maternal genome donors of the 2*n* = 4*x* = 30 allotetraploid cytotype (Catalán *et al.* 2012, 2016b). The 2*n* = 2*x* = 20 lineage was estimated to be older and mutated significantly faster than the 2*n* = 2*x* = 10 lineage. Taking into account all the phenotypic, cytogenetic and molecular differences among the three cytotypes, their taxonomic separation into three different species was first reported by Catalán *et al.* (2012) who described the two novel species: *B. stacei* (2*n* = 2*x* = 20) and *B. hybridum* (2*n* = 4*x* = 30).

*Brachypodium distachyon*, *B. stacei* and *B. hybridum* are distributed by the entire circum-Mediterranean zone (Schippmann 1991; Garvin *et al.* 2008; Catalán *et al.* 2012; López-Álvarez *et al.* 2012). They can grow in different environments with different abiotic and biotic conditions that could be associated with adaptive natural genetic variation (Garvin *et al.* 2008; Manzaneda *et al.* 2012). *Brachypodium distachyon* and *B. hybridum* populations are widespread in the Mediterranean region and largely overlap (López-Álvarez *et al.* 2012). *Brachypodium stacei* populations have been detected in both western and eastern Mediterranean regions and also in SW Asian-Middle East region. *Brachypodium distachyon* grows in higher, cooler and wetter places; *B. stacei* in lower, warmer and drier places; and *B. hybridum* in places with intermediate climatic features. *Brachypodium hybridum* had the largest niche overlap with its parent niches, but a similar distribution range and niche breadth (López-Álvarez *et al.* 2012, 2015). The distribution of *B. distachyon* and *B. hybridum* species is geographically structured throughout its range in the Iberian Peninsula, and is associated with aridity gradients, i.e. *B. distachyon* commonly appears in the east and north of the Iberian Peninsula, whereas in the south is either restricted to montane areas at mid- or high elevations, or in populations located near the coast, frequently in wet habitats in which summer drought is attenuated (Manzaneda *et al.* 2012). Contrastingly, *B. hybridum* grows across the Iberian Peninsula at low to mid-elevations, in NE and NW Spain (all places except the very humid North) inhabiting dry environments in which a predictable summer drought period exists (Manzaneda *et al.* 2012; López-Álvarez *et al.* 2012, 2015). Recently, Marques *et al.* (2017) have found a higher genetic diversity in eastern Iberian populations of *B. distachyon* occurring in basic soils suggesting that these populations could be less constrained than those occurring in western areas of the Iberian Peninsula where the soils are more acidic and accumulate toxic Al ions. This result suggests that the western Iberian acidic soils might prevent the establishment of Al-sensitive *B. distachyon* populations, potentially causing the existence of more genetically depauperate individuals.

Genotypes growing in acidic soils may have multiple origins but the possibility of a common origin cannot be excluded. In the latter case, one would expect that the Al-tolerant genotypes would share common neutral molecular markers and were more genetically related.

These species (*B. distachyon*, *B. stacei* and *B. hybridum*) show a great similarity for most external traits, which explains that they were classified with the same botanical name, *B. distachyon*. Their correct classification using only phenotypic and metabolic traits is feasible though laborious (Catalán *et al.* 2016a; López-Álvarez *et al.* 2017). Hence, it is important to develop taxonomic methods based in the use of DNA genetic markers that facilitate to identify an organism as belonging to a particular species and to assign the new collected accessions to the right species (López-Álvarez *et al.* 2012). This is specially demanded for this group of species since other quick procedures, as flow cytometry, cannot distinguish clearly between *B. distachyon* and *B. stacei* (Draper *et al.* 2001; Catalán *et al.* 2012; Manzaneda *et al.* 2012), and only the time-consuming cytological analysis is a fully reliable alternative.

All the accessions used in this work were previously classified as Al-sensitive or Al-tolerant based on the relative root regrowth after Al stress using different histochemical methods described in Contreras *et al.* (2014). In addition, this study detected an insertion in the promoter region of the *BdALMT1* gene in all *B. hybridum* and *B. distachyon* Al-tolerant accessions. The *BdALMT1* gene is an orthologous of the Al-tolerance gene *TaALMT1* of wheat and probably has a similar function. These genes, belonging to the Al-activated malate transporter family, are involved in Al-tolerance in multiple species (Delhaize *et al.* 2012b). Contreras *et al.* (2014) demonstrated that the exudation of citrate and malate was induced only in the roots of Al-tolerant allotetraploid *B. hybridum* samples and of the tolerant diploid accession ABR8 of *B. distachyon*. These authors also showed that the transcription of *BdALMT1* gene was induced by Al in the tolerant accessions ABR8 (*B. distachyon*) and CB (*B. hybridum*), but nor in the Al-sensitive accessions ABR1 (*B. distachyon*) and GRA 788 (*B. hybridum*). The higher levels of *BdALMT1* transcript found in the former could be related with the insertion present in the promoter of *BdALMT1* gene and related with the induction and the high expression detected in Al-tolerant accessions of *B. hybridum* and *B. distachyon*. Therefore, to characterize the natural diversity of annual *Brachypodium* species in relation to the Al-tolerance and their potential common origin from acidic soil regions and to provide a valuable new tool to *Brachypodium* researchers, we have analysed a diverse collection of *B. distachyon* (one tolerant and 51 sensitive accessions), *B. stacei* (nine sensitive accessions) and *B. hybridum* (10 tolerant and 23 sensitive accessions) accessions that came mainly from the Iberian Peninsula using new SSR and ISSR neutral molecular markers and the amplification of the *BdALMT1* upstream-intron 1 region.

## Methods

### Plant materials

**Figure 1.**
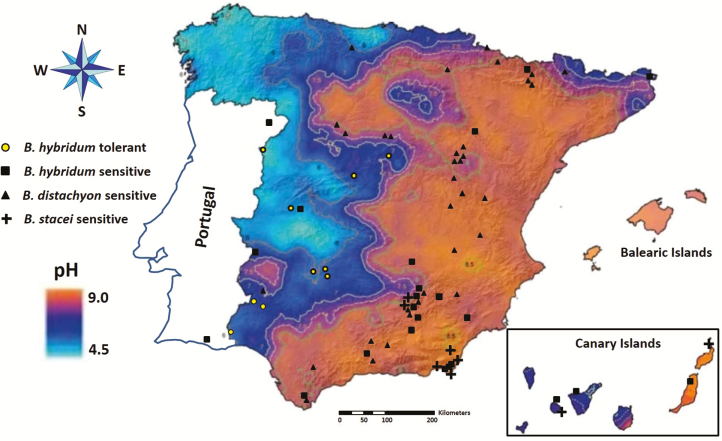
Map of Spain and Portugal including the soil pH data from Fernández *et al.* (1971), Rita and Rossello (1989) and López and Grau (2004). The location of the different *Brachypodium distachyon* and *B. stacei* diploid accessions and *B. hybridum* allotetraploid accessions is indicated.

Fifty-two diploid accessions (2*n* = 2*x* = 10) of *B. distachyon*, nine diploid accessions (2*n* = 2*x* = 20) of *B. stacei* and 33 allotetraploid accessions (2*n* = 4*x* = 30) of *B. hybridum* were studied **[see Supporting Information—Table S1]**. The sampled populations from the Iberian Peninsula and Balearic and Canary Islands cover most of the ranges of distribution, elevation and types of climate and habitat in which these species grow (**see Supporting Information—Table S1**; Fig. 1). All plants were cultivated in a growth chamber kept at 22 °C with a 16-h photoperiod.

### Selection of pentanucleotide SSR loci, DNA extraction, SSR multiplex PCR and ISSR-PCR amplifications

Seventeen pentanucleotide SSR loci were selected from the five different chromosomes of *B. distachyon* using the sequence of Bd21 accession (The International *Brachypodium* Initiative 2010) **[see Supporting Information—Fig. S1]** and the Phobos 3.3.1.1 software (Mayer 2006–2010, http://www.rub.de/spezzoo/cm/cm_phobos.htm). In order to obtain amplification products with small sizes (between 100 and 450 bp) easy to separate using capillary electrophoresis, pentanucleotide SSRs showing a small number of repeat units (between four and nine) in the IBI Bd21 reference genome were selected.

In total, 2009 pentanucleotide SSRs were identified using the Bd21 sequence, Phobos 3.3.1.1 software and the cut-off value of four repeats. Five hundred and fifty-four SSRs were detected on chromosome 1 (number of repeats ranged from 4 to 16), 446 on chromosome 2 (repeats between 4 and 13), 456 on chromosome 3 (repeats between 4 and 12), 369 on chromosome 4 (repeats between 4 and 20) and 184 on chromosome 5 (repeats between 4 and 11). Several combinations of different SSRs were tested to find compatible multiplex PCR amplifications using the MPprimer software. The two sets of compatible multiplex PCR developed were based on: (i) ease of score, (ii) a compatible allele size range and (iii) similar optimal reaction conditions. Three SSRs were located on chromosome 1, four on chromosome 2, three on chromosome 3, three on chromosome 4 and four on chromosome 5 **[see Supporting Information—Fig. S1]**.

Two different multiplex PCR assays were designed using the MPprimer software (Shen *et al.* 2010). The multiplex PCRs amplified nine and eight different SSRs (Sets 1 and 2, respectively; **see Supporting Information—Table S2**). Different fluorescent dyes (TAM, HEX, AT565, FAM) were used for primer labelling to discriminate co-amplified products either by size or by colour **[see Supporting Information—Table S2]**.

DNA was extracted from ~0.05 g of young leaf tissue with the DNAeasy plant mini kit (Qiagen). PCR-Multiplex Set 1 reactions were carried out with 4.7 μL genomic DNA (5 ng μL^−1^), 7 μL *Taq* PCR 2× Master Mix from Qiagen and 2.3 μL of nine primer pairs **[see Supporting Information—Table S3]**. PCR-Multiplex Set 2 reactions were carried out with 4.7 μL genomic DNA (5 ng μL^−1^), 7 μL *Taq* PCR 2× Master Mix from Qiagen, 0.5 μL free nuclease water and 1.8 μL of eight primer pairs **[see Supporting Information—Table S3]**. Finally, 0.25 μL of the PCR-Multiplex products were mixed with 13.75 μL Mili-Q water and the GeneScan™-500 LIZ® dye size standard and run on an ABI3730xl (Applied Biosystems). The PCRs for Set 1 were carried out in a final volume of 14 μL on a MJ Research PTC-100 thermocycler with a thermal profile consisting of 2 min initial temperature denaturation step at 95 °C followed by 35 cycles of 45 s at 95 °C, 45 s at 58 °C and 2 min at 72 °C. A final 72 °C extension step of 6 min was included to promote non-templated nucleotide addition at the 3′ end of the PCR products. The PCRs for Set 2 were carried out also in a final volume of 14 μL with a thermal profile consisting of 2 min initial temperature denaturation step at 95 °C followed by 40 cycles of 45 s at 95 °C, 45 s at 57 °C and 3 min at 72 °C. A final 72 °C extension step of 6 min was included. The Peak Scanner Software v 1.0 (Applied Biosystems) was used for the analysis of the SSR products.

Seven ISSR primers (Table 1) were selected from the set of 100 ISSR primers (UBC set # 9) from the Biotechnology Laboratory, University of British Columbia, Vancouver, Canada. These ISSR selected primers were used to amplify the genomic DNA of 52 diploid accessions (2*n* = 2*x* = 10) of *B. distachyon* and 33 allotetraploid accessions (2*n* = 4*x* = 30) of *B. hybridum***[see Supporting Information—Table S1]**. For each primer, a 12 μL amplification reaction was prepared containing: 3.6 μL of DNA (5 ng μL^−1^), 6 μL of *Taq* PCR 2× Master Mix from Qiagen and 2.4 μL of primer (5 mM). PCR amplifications were performed in a MJ Research PTC-100 thermal cycler system programmed with an initial denaturation step at 94 °C (5 min), followed by 45 amplification cycles of 94 °C (30 s), 52 °C (45 s) and 72 °C (2 min) and with a final extension step at 72 °C (10 min) (Zietkiewicz *et al.* 1994). The amplification products were separated by capillary electrophoresis using a QIAxcel instrument with a QIAxcel cartridge (Qiagen). The results were analysed using the BioCalculator Software (Qiagen). DNA amplifications with each ISSR primer were repeated at least three times. The amplification bands were considered reproducible only after their observation in the three replicates. Only clear and intense bands were scored.

**Table 1.** ISSR markers analysed. Total number of bands (TNB), percentage of polymorphic bands (P %) at 99 %, number of different genotypes or isolates identified (NG), resolving power (Rp) and number of exclusive bands (NPB) obtained with each ISSR primer. R = A, G; Y = C, T.

Diploid lines of *Brachypodium distachyon* (2*n* = 2*x* = 10)						
Primer	Sequence	TNB	P % (99 %)	NG	Rp	NPB
811	(GA)_8_C	21	95.2	46	7.3	0
834	(AG)_8_YT	17	100	34	7.9	2
835	(AG)_8_YC	15	86.7	40	5.4	1
842	(GA)_8_YG	22	95.4	44	6.9	1
844	(CT)_8_RC	15	93.3	30	4.1	3
846	(CA)_8_RT	15	80	13	2.1	4
855	(AC)_8_AYT	11	100	22	3.1	2
Total		116	93.1	229	37	13
Allotetraploid lines of *Brachypodium hybridum* (2*n* = 4*x* = 30)						
Primer	Sequence	TNB	P % (99 %)	NG	Rp	NPB
811	(GA)_8_C	14	100	28	9.1	0
834	(AG)_8_YT	21	100	29	10	1
835	(AG)_8_YC	22	95.6	32	9.8	1
842	(GA)_8_YG	22	100	28	11.2	3
844	(CT)_8_RC	20	100	31	10.1	2
846	(CA)_8_RT	18	100	26	8.4	2
855	(AC)_8_AYT	17	95.6	32	7.8	3
Total		139	98.6	206	66.5	12

### Upstream-exon 1 region of the *ALMT1* gene for species discrimination

The upstream-exon 1 region of the *ALMT1* gene was selected for this purpose because we have previously detected differences in this region between the *B. distachyon* and *B. stacei* (Contreras *et al.* 2014). Two insertions were detected in *B. stacei* that were absent in *B. distachyon*. In order to distinguish the samples from *B. distachyon*, *B. stacei* and *B. hybridum*, a specific pair of primers was designed from the conserved sequences of *B. distachyon* and *B. stacei ALMT1* genes (Contreras *et al.* 2014). The forward primer (5′-CCGAATACACATCGACCTCCTCAT-3′) was located in the upstream region and the reverse primer (5′-CCCGAGCCCGAGGACCAACGAG-3′) in the exon 1 of these *ALMT1* genes. A 12 μL amplification reaction was prepared containing: 3.6 μL of DNA (5 ng μL^−1^), 6 μL of *Taq* PCR 2× Master Mix from Qiagen and 1.2 μL of each primer (5 mM). The amplification with these primers was carried out in a MJ Research PTC-100 thermal cycler system programmed with an initial denaturation step at 95 °C (3 min), followed by 26 amplification cycles of 95 °C (20 s), 55 °C (30 s) and 72 °C (1 min) and with a final extension step at 72 °C (7 min). Amplified products were analysed by electrophoresis in 1.2 % agarose gels.

### Diversity index, genetic relationships, principal component analyses and Mantel test

The total number of ISSR bands (amplified products, TNB), percentage of polymorphic bands (P %) at 99 %, number of different genotypes (NG), resolving power (Rp) and number of private bands (NPB) obtained with each primer were estimated. These estimations were carried out independently for *B. distachyon* and *B. hybridum* species. In *B. stacei* only 5 of the 17 pair of SSR primers selected gave good amplification results using the two multiplex PCR developed. Due to this reason, the nine samples of *B. stacei* were not included in the genetic relationship studies.

According to Prevost and Wilkinson (1999), the Rp of an ISSR primer was calculated as Rp = ΣIb where Ib (band informativeness) takes the value of: 1 − [2 × (0.5 − *p*)], *p* being the frequency of the accessions containing the band.

The polymorphic information content (PIC) of a SSR locus was estimated as PIC = 1 − Σpi2, pi being the frequencies of the different alleles detected in this locus. Each ISSR locus has only two alleles (presence or absence of one band). The PIC of an ISSR locus was estimated as PIC = 2*p*(1 − *p*), *p* being the frequency of the accessions with the band (or the frequency of the positive allele) and (1 − *p*) the frequency of the accessions where the band was absent (or the frequency of the null allele). Therefore, both methods to estimate PIC are equivalent and comparable.

The establishment of genetic relationships using pentanucleotide SSRs from *B. distachyon* and *B. hybridum* accessions was carried out with the Phylip 3.6 software (Felsenstein 2001), two genetic distances: Nei 72 (Nei 1972) and Cavalli-Sforza and Edwards (1967) chord distance, and two clustering methods: UPGMA (unweighted pair group method with arithmetic mean) and NJ (neighbor-joining). Bootstraps with 1000 replicates were performed to test the robustness of the genetic trees. The dendrograms were drawn using TreeView 1.5 (Page 1996) and the software FigTree 1.4.0 (http://tree.bio.ed.ac.uk/software/figtree/).

Genetic relationships were also analysed using ISSR markers and the NTSYSpc 2.0 software (Rohlf 1993). Three different similarity coefficients, Dice, Jaccard and simple matching (SM), and the UPGMA clustering method were used. To determine the confidence limits of dendrograms, bootstrap tests (1000 replications) were performed using the WinBoot software (Yap and Nelson 1996). Additional trees were obtained with Phylip 3.6 program, RESTDIST distance, NJ and 1000 bootstrap replicates. Finally, other genetic trees were obtained (1000 bootstrap replicates) using the parsimony methods and the BLMARKER and DOLLOP polymorphism included in the BIRCH 2.96 software (Fristensky 2007) because these programs are indicated to dominant markers like the ISSRs.

In order to analyse the genetic relationships, principal component analyses (PCAs) were performed with the NTSYSpc 2.0 software using both the SSR and ISSR marker data sets. Nei 72 and Cavalli-Sforza and Edwards chord distances were used for SSR data and Dice, Jaccard and SM coefficients matrices for ISSR data (Rohlf 1993).

To evaluate the potential correlation degree between pairwise geographical distances between the *B. distachyon* and *B. hybridum* accessions and the pairwise distance matrices based on SSR (Nei 72 distance) and ISSR (Dice, Jaccard and SM coefficients) data, independent Mantel test (Mantel 1967) for *B. distachyon* and *B. hybridum* accessions was performed using the NTSYSpc 2.0 software. The number of permutations was 250 in all cases.

Soil pH values were retrieved from López and Grau (2004) and the map of soil pH from Rodríguez *et al.* (2009).

## Results

### Pentanucleotide microsatellites and inter-microsatellite markers data

The 17 primer pairs used to study the SSR loci were designed from the genome sequence of *B. distachyon* Bd21 accession. All primer pairs gave good results in *B. distachyon* and *B. hybridum*, whereas only five pairs produced robust amplification products in *B. stacei*. This suggests differences in the genomic sequences of the priming sites between the two diploid species. We have tested several different PCR conditions in order to develop the two multiplex PCR analyses and the conditions indicated in methods gave the best results.

The number of pentanucleotide SSR loci detected in the five chromosomes of *B. distachyon* was in agreement with the size of each chromosome. The large chromosome 1 presented the highest number of SSR loci (554) and the small chromosome 5 showed the lowest number of SSR loci (184).

The total number of alleles detected for the selected 17 SSR loci of the *B. distachyon* accessions was 55, with an average number of alleles per locus of 3.23. Two of these loci (Bd1GSSR_186 and Bd3GSSR_357) showed six alleles and one locus was monomorphic (Bd5GSSR_371) (Table 2). The five SSR loci that could be analysed in *B. stacei* were all monomorphic in nine accessions examined. Therefore, the total number of alleles detected in *B. stacei* was five (Table 2). The sizes of the alleles observed in loci amplified in the nine lines of *B. stacei* were always different from the sizes of the alleles of the orthologous loci of *B. distachyon* (Table 2). In *B. hybridum*, these five primer pairs produced invariably two PCR products (two alleles), one of them with the allele size found in *B. stacei*. The other allele, with a different size, was then assigned to the *B. distachyon* genome of the allotetraploid. In total, the number of SSR loci analysed in *B. hybridum* accessions was 22; 17 of them were amplified from the genome of *B. distachyon* and the other five SSR loci were amplified from the genome of *B. stacei*. The 17 SSR loci of *B. hybridum* amplified from the *B. distachyon* genome showed a total of 43 alleles, with an average number of 2.53 alleles per locus. One of these loci had five alleles (Bd3GSSR_357) and two were monomorphic (Bd5GSSR_371 and Bd1GSSR_174) (Table 2).

**Table 2.** Pentanucleotide SSR markers used to analyse population diversity in *Brachypodium distachyon* (*Bd*), *B. stacei* (*Bs*) and *B. hibridum* (*Bh*). The exclusive alleles detected in the tolerant *B. distachyon* line ABR8 are indicated in a separated column (Tolerant *Bd*). The exclusive alleles of the 10 tolerant *B. hybridum* lines are indicated in another column (Tolerant *Bh*). The exclusive alleles detected in all tolerant *B. hybridum* lines (frequency 1) and absent in all sensitive *B. hybridum* lines are indicated by an asterisk. The sizes of the different alleles are indicated in base pairs between brackets.

SSR	Alleles of *Bd*	Tolerant *Bd* (private)	Alleles of *Bh*	Tolerant *Bh* (private)	Alleles of *Bs*
Bd3GSSR_145	4 (140, 145, 150, 155)	1 (140)	4 (135, 140, 145, 155)	2 (135*, 155)	
Bd1GSSR_186	6 (160, 165, 170, 175, 180, 185)	1 (160)	3 (170, 175, 185)		
Bd2GSSR_212	2 (210, 215)		4 (195, 205, 210, 215)	1 (195)	
Bd2GSSR_212 (*Bs*)	-		1 (185)		1 (185)
Bd3GSSR_261	3 (245, 250, 260)		2 (250, 260)		
Bd2GSSR_273	4 (260, 265, 270, 280)		2 (260, 265)		
Bd5GSSR_311	3 (304, 309, 319)	1 (319)	3 (304, 309, 319)		
Bd5GSSR_371	1 (369)		1 (369)		
Bd4GSSR_399	4 (384, 389, 394, 399)		3 (384, 394, 399)	1 (384*)	
Bd4GSSR_399 (*Bs*)	-		1 (374)		1 (374)
Bd2GSSR_430	4 (421, 426, 431, 436)		4 (421, 431, 436, 446)	1 (446*)	
Bd5GSSR_128	2 (113, 123)		2 (113, 123)	1 (113*)	
Bd5GSSR_128 (*Bs*)	-		1 (104)		1 (104)
Bd1GSSR_174	3 (160, 165, 170)		1 (164, 165)	1 (164*)	
Bd1GSSR_174 (*Bs*)	-		1 (164)		1 (164)
Bd5GSSR_187	3 (175, 180, 185)		3 (180, 185, 190)	1 (190)	
Bd4GSSR_219	2 (212, 217)		3 (192, 212, 217)	1 (192*)	
Bd4GSSR_272	2 (263, 268)		1 (263, 268)		
Bd4GSSR_272 (*Bs*)	-		1 (253)		1 (253)
Bd2GSSR_291	3 (278, 288, 293)		3 (278, 283, 288)	1 (283)	
Bd1GSSR_303	3 (298, 303, 308)		3 (293, 298, 303)	1 (298*)	
Bd3GSSR_357	6 (332, 342, 347, 352, 357, 362)	1 (332)	5 (327, 332, 342, 347, 352)	1 (327*)	

Four private SSR alleles were found in the ABR8 *B. distachyon*-tolerant accession and 12 in the *B. hybridum*-tolerant accessions, 7 of them being present in all the 10 *B. hybridum*-tolerant accessions (frequency 1) (Table 2).

With the seven ISSR primers selected the TNB was of 116 and 139 in *B. distachyon* and *B. hybridum* accessions, respectively, with an average of 16.6 (*B. distachyon*) and 19.6 (*B. hybridum*) bands per primer. The maximum number of amplified products was 22 (primer 835 in *B. hybridum* and primer 842 in both species) and the minimum was 11 (primer 855 in *B. distachyon*) (Table 1). The Rp of the seven ISSR primers in *B. distachyon* accessions ranged from 2.1 for primer 846 to 7.9 for primer 834. In the case of *B. hybridum* accessions, Rp ranged from 7.8 for primer 855 to 11.2 for primer 842. The primer 811 was able to distinguish 46 different *B. distachyon* genotypes and the 835 and 855 primers were able to distinguish 32 different *B. hybridum* genotypes. The NPB observed in all accessions of *B. distachyon* that were absent in all accessions of *B. hybridum* was 13, whereas the NPB present in all accessions of *B. hybridum* that were absent in all accessions of *B. distachyon* was 12 (Table 1). In addition, the NPB observed in the ABR8 tolerant *B. distachyon* accession and absent in the remainder 51 sensitive *B. distachyon* accessions was 11. Finally, the NPB observed in all tolerant *B. hybridum* accessions and absent in all sensitive *B. hybridum* accessions was 7 and the NPB present in all sensitive *B. hybridum* accessions and absent in all tolerant *B. hybridum* accessions was 4.

### Species discrimination based on upstream-exon 1 region of *ALMT1* gene and in SSR loci

The upstream-exon 1 region of the *B. stacei ALMT1* gene has several insertions compared with the same gene region of *B. distachyon* (Contreras *et al.* 2014). The amplification of this region with a specific pair of primers in the genomic DNA from *B. distachyon* and *B. stacei* diploid cytotypes produces a DNA fragment with a different size in each species, 506 bp in *B. distachyon* and 629 bp in *B. stacei***[see Supporting Information—Fig. S2]**. However, the amplification of this *ALMT1* region in the 33 *B. hybridum* accessions analysed in this study produced simultaneously two fragments with the same sizes (506 and 629 bp) of the amplification products observed in *B. stacei* and *B. distachyon***[see Supporting Information—Fig. S2a]**.

Among the five SSR loci amplified in *B. stacei*, Bd2GSSR_212, Bd4GSSR_399, Bd5GSSR_128 and Bd4GSSR_272 SSR showed one allele with clearly different size in *B. stacei* and *B. distachyon* and two alleles in *B. hybridum* (Table 2). These four SSR loci can then distinguish the three different species by using either capillary electrophoresis or agarose gels. The Bd1GSSR_174 locus can be used also to distinguish the three species but only with capillary electrophoresis. The allele of Bd1GSSR_174 with a size of 164 bp observed in *B. stacei* had 1 bp difference with the 165 bp allele of *B. distachyon*. Taking into account that this is a pentanucleotide SSR, the size detected in the nine *B. stacei* accessions studied in this work presented a deletion of 1 bp in the amplification product of this SSR locus.

### Genetic relationships

**Figure 2.**
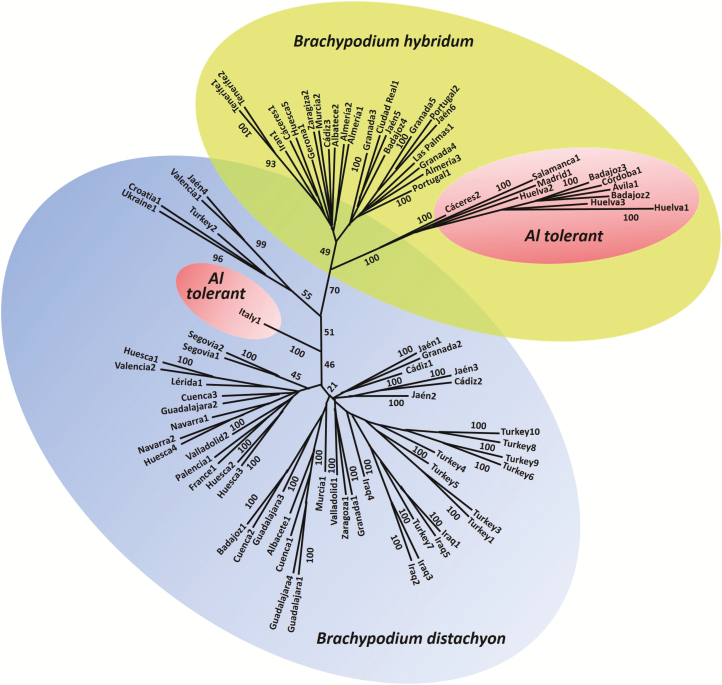
Dendrogram showing relationships of *Brachypodium distachyon* and *B. hybridum* accessions based on SSR markers using the Cavalli-Sforza and Edwards distance, NJ grouping method and 1000 bootstrap replicates. The 33 *B. hybridum* accessions are clearly separated from the *B. distachyon* accessions. Also, the 10 *B. hybridum*-tolerant accessions are grouped in a different cluster (big pink ellipse). The tolerant *B. distachyon* accession ABR8 (Italy, small pink ellipse) is also separated from the other diploid accessions. The original sources of the accessions are indicated in **Supporting Information—Table S1**.

**Figure 3.**
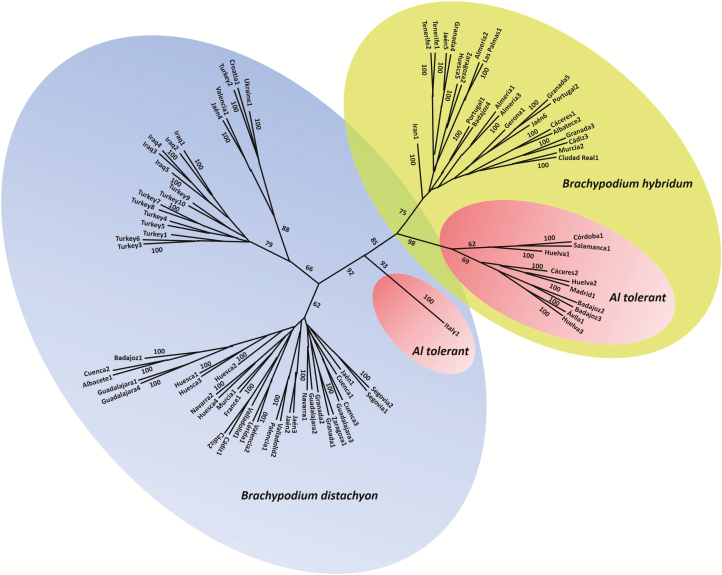
Dendrogram showing relationships of *Brachypodium distachyon* and *B. hybridum* accessions based on ISSR markers using parsimony method (BIRCH 2.96 software) and 1000 bootstrap replicates. The 33 *B. hybridum* accessions are clearly separated from the *B. distachyon* accessions. Also, the 10 *B. hybridum*-tolerant accessions are grouped in a different cluster (big pink ellipse). The tolerant *B. distachyon* accession ABR8 (Italy, small pink ellipse) is also separated from the other *B. distachyon* accessions. The original sources of the accession are indicated in **Supporting Information—Table S1**.

Two dendrograms were obtained using the SSR markers with Phylip 3.6 software, the Nei 72 and Cavalli-Sforza and Edwards chord distances, and NJ clustering method. The two dendrograms obtained showed a very similar structure with the same main clusters. Due to this reason we selected the dendrogram corresponding to chord distance and NJ method (Fig. 2). The other dendrogram can be consulted in **Supporting Information—Fig. S3**. An ISSR-based dendrogram obtained using the BIRCH 2.96 software and a parsimony method was similar to the SSR trees (Fig. 3). With the ISSR markers, additional dendrograms were obtained utilizing the NTSYSpc software, three different similarity coefficients (Dice, Jaccard and SM) and the UPGMA method, all of them showing the same structure with the same clusters **[see Supporting Information—Fig. S4]**. This result is in agreement with the high correlation coefficients (*r*) estimated with the Mantel test using the pairwise genetic matrices **[see Supporting Information—Table S4]**.

The ABR8 *B. distachyon* accession (2*n* = 2*x* = 10) from Siena, a region of Italy with acidic soils, was Al-tolerant, whereas the remaining 51 *B. distachyon* accessions were Al-sensitive. Two of these 51 sensitive accessions grow in regions with acidic soils. The nine Al-sensitive *B. stacei* accessions (2*n* = 2*x* = 20) came from Spanish regions with basic soils. Finally, the 10 Al-tolerant *B. hybridum* accessions (2*n* = 4*x* = 30) were from areas of Spain with acidic soils, whereas the Al-sensitive *B. hybridum* accessions analysed (20 from Spain, two from Portugal and one from Iran) came mostly from regions with basic soils, only four Al-sensitive *B. hybridum* accessions came from acidic soils, two from Portugal (Tras-os-Montes and Algarve) and other two from Spain (Extremadura) (**see Supporting Information—Table S1**; Fig. 1).

**Figure 4.**
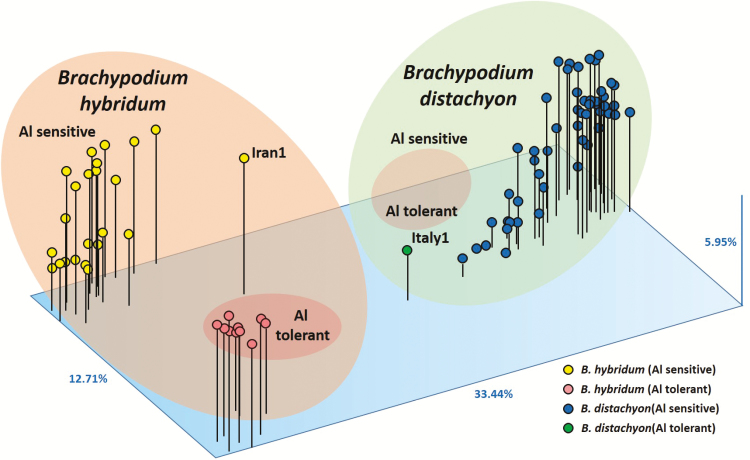
Bidimensional plot of *Brachypodium distachyon* and *B. hybridum* accessions revealed by PCA based on ISSR data using the SM coefficient. The original sources of the accessions are indicated in **Supporting Information—Table S1**.

All dendrograms showed the same main clusters independently of the markers and methods used. The diploid *B. distachyon* and allotetraploid *B. hybridum* accessions were grouped into two different clusters with a bootstrap of 70 (SSRs) or 85 (ISSRs). The tolerant *B. distachyon* ABR8 accession from Italy was separated from the sensitive *B. distachyon* samples in the ISSR dendrograms (bootstrap 100) but no clearly separated in the SSR dendrograms (bootstrap 100). Moreover, the samples from Turkey and Iraq were separated in a different subgroup in the ISSR dendrograms but together with two samples from Spain (Valencia1 and Jaen4). However, the samples from Turkey and Iraq were not clearly separated in a different subgroup in the SSR dendrograms. The tolerant *B. hybridum* samples were grouped together in a subcluster and the sensitive *B. hybridum* accessions in another subcluster with a bootstrap of 100 (SSRs) or 98 (ISSRs). The PCAs carried out with ISSRs using the three similarity coefficients (Dice, Jaccard and SM) yielded similar results with the first three Eigen vectors accounting for 52.10 % of the total variance (33.44 % the first vector, 12.71 % the second vector and 5.95 % the third vector) **[see Supporting Information—Fig. S5]**. A clear distinction among the different accessions (Spanish and Iranian, sensitive and tolerant allotetraploids of *B. hybridum* and sensitive and tolerant diploids of *B. distachyon*) was observed in the PCAs with ISSRs (Fig. 4). The two samples from Portugal were sensitive and they were grouped with the Spanish sensitive allotetraploid samples. The 33 allotetraploid accessions are clearly separated from the diploid ones. Also, the 10 allotetraploid-tolerant accessions are situated in a different group. The tolerant diploid accession ABR8 (Italy) is also detached from the other diploid accessions. The SSR PCAs also split the diploid from the allotetraploid accessions.

The Mantel tests performed between pairwise geographical and genetic distance matrices (SSR markers with Nei 72 and Cavalli-Sforza and Edwards distances, ISSR markers with SM, Dice and Jaccard coefficients) corresponding to *B. distachyon* accessions determined that none of the correlation coefficients obtained are statistically significant **[see Supporting Information—Table S4]**. Moreover, significant correlation is not found when the geographical distribution of the Spanish *B. hybridum* accessions is compared with the genetic distance matrices (SSR markers with Nei 72, ISSR markers with SM, Dice and Jaccard). The correlations obtained when different pairwise genetic distance matrices are compared were significant **[see Supporting Information—Table S4]**.

## Discussion

### Quantification of genetic variability

The average PIC obtained with the pentanucleotide SSR loci was higher in *B. hybridum* accessions (0.5) than in *B. distachyon* ones (0.42). This significant difference cannot be clearly attributed to the allotetraploid genome constitution of *B. hybridum* because the five loci that came from *B. stacei* were monomorphic. Giraldo *et al.* (2012) obtained an average PIC of 0.55 with four SSR loci (di- and tri-nucleotides) in 64 Spanish lines of *B. distachyon*. Vogel *et al.* (2009) analysed six accessions of *B. distachyon* and reported that the SSR loci with more number of repeats (13 or more) were more polymorphic (3.2 alleles per locus) than SSR loci with less number of repeats (five, with 1.5 alleles per locus) in six accessions of *B. distachyon*. In the present study, the mean number of alleles per locus has been of 3.2 and 2.5 for *B. distachyon* and *B. hybridum*, respectively. Taking into account the much larger sample size (52 diploid accessions), those values would be in agreement with the lower number of repetitions (from 4 to 9) of the SSR loci analysed here.

All accessions of *B. distachyon* were homozygotes in the 17 loci studied, each SSR locus showing only one allele in each accession. A high homozygosity was also obtained by Vogel *et al.* (2009) using 43 SSR loci and 187 diploid accessions of *B. distachyon* and by Marques *et al.* (2017) using 10 SSR loci and 14 populations of *B. distachyon* sampled across the Iberian Peninsula. This suggests a high homozygosity and reflects that *B. distachyon* is mainly a selfing species. When the *B. hybridum* accessions were analysed, all of them seemed to homozygotes not only for the 17 SSR loci that came from *B. distachyon*, but also for the 5 SSR loci that came from *B. stacei*. The presence of two amplification products (two alleles) for each of these 5 SSR primers in *B. hybridum* could lead to infer that the plants were heterozygotes. However, the finding that their respective allele sizes invariably correspond to one allele found in *B. distachyon* and the other allele present in the *B. stacei* accessions examined makes it very unlike such conclusion. The most probably explanation is that the *B. hybridum* accessions are homozygotes in two different orthologous loci located in equivalent chromosome regions of the two different genomes from *B. distachyon* and *B. stacei* present in *B. hybridum*. The remaining 12 SSR primers, that only amplified in *B. distachyon* genome but not in *B. stacei* genome, showed a single amplification product in the *B. hybridum* accessions analyzed. These results suggest that *B. hybridum* could be a primarily selfing species and also evidence its allotetraploid origin. Similar conclusions were drawn from the SSR analysis of Giraldo *et al.* (2012) on a collection of 64 *B. distachyon*, two *B. stacei* and 12 *B. hybridum* Spanish accessions.

As expected, the diversity estimators obtained using ISSR markers (Table 2) were significant and higher in *B. hybridum* allotetraploids than in *B. distachyon* diploids. The percentage of polymorphic bands in *B. distachyon* (93.1 %) and *B. hybridum* (98.6 %) was higher than the estimated in barley (83 %), an autogamous cultivated species (Fernández *et al.* 2002), and among different cultivars of the allogamous cultivated *Secale cereale* (82 %) (Matos *et al.* 2001). Barley and rye are diploid domesticated species and probably these characteristics could explain their lower percentage of polymorphic bands compared to *B. distachyon*. Moreover, when this percentage was estimated using random amplified polymorphic DNA (RAPD) markers in barley (Fernández *et al.* 2002) and rye (Matos *et al.* 2001), the values were also lower than the obtained in *B. distachyon* using ISSR markers. The average PIC calculated with the seven ISSR primers was higher in *B. hybridum* (0.32) than in *B. distachyon* (0.22) as it was observed using the SSR loci. The average PIC estimated with ISSR markers was higher in Al-sensitive (0.3) *B. hybridum* samples (from the eastern areas of the Iberian Peninsula) than in Al-tolerant (0.1) *B. hybridum* accessions (from western areas of the Iberian Peninsula). The average number of alleles per locus calculated using SSR markers was also higher in Al-sensitive (2.3) *B. hybridum* than in Al-tolerant (1.4) *B. hybridum* accessions. This result agrees with data obtained by Marques *et al.* (2017) in *B. distachyon* populations from the Iberian Peninsula. Similarly to *B. distachyon*, the acidic soils might prevent the establishment of Al-sensitive *B. hybridum* populations, probably causing the existence of more genetically depauperate individuals.

### DNA barcoding identification of *B. distachyon*, *B. stacei* and *B. hybridum* accessions

Eleven private ISSR markers were observed in the *B. distachyon*-tolerant ABR8 accession and seven in the 10 *B. hybridum*-tolerant samples that were absent in the allotetraploid-sensitive plants **[see Supporting Information—Table S5]**. These new neutral markers could be used, in addition to the earlier described insertion present in the promoter region of the *BdALMT1* gene (Contreras *et al.* 2014), as molecular tools to distinguish Al-tolerant from Al-sensitive accessions in future studies after testing across more accessions of these two species to confirm their respective diagnostic value.

In order to get an easy DNA barcoding method to discriminate among *B. distachyon*, *B. stacei* and *B. hybridum* species, we have developed a very effective molecular marker based on the existence of several indels between the upstream-exon 1 sequences from the *BdALMT1* (*B. distachyon*) and *BsALMT1* (*B. stacei*) genes. This new species-specific marker is based on a simple PCR with a specific pair of primers, which is usually a quick and reliable method not prone to failure pending of PCR conditions. The indels that differentiate the promoter region (upstream) and exon 1 of the *ALMT1* gene of *B. stacei* and *B. distachyon* could explain the absence of Al-tolerant *B. stacei* accessions.

In addition, we have obtained four SSR loci (Bd2GSSR_212, Bd4GSSR_399, Bd5GSSR_128 and Bd4GSSR_272) that can also distinguish among the three species using agarose gels because the *B. hybridum* samples show two different alleles with different sizes, one of them from *B. distachyon* and another from *B. stacei***[see Supporting Information—Fig. S2]**. A similar species-specific profile has been described for other SSR loci (ALB165, ALB311, BD330 and R2-3) by Giraldo *et al.* (2012). However, we have used SSR pentanucleotide loci, which makes more easy to distinguish the diploid species alleles by their size.

López-Álvarez *et al.* (2012) published a very good DNA barcoding method to discriminate these species using three loci: one maternally inherited plastid trnLF region, the nuclear multicopy ribosomal internal transcribed spacer ITS region and the nuclear single-copy GIGANTEA gene (GI). This method, based on PCR amplification and DNA sequencing of specific DNA fragments of these three loci, provides highly stable and reliable data. The sequences were indeed tested across a large sampling of *B. distachyon*, *B. satcei* and *B. hybridum* individuals distributed across the whole circum-Mediterranean region (Lopez-Alvarez *et al.* 2012). However, it is quite time-consuming and the identification of *B. hybridum* is only possible using simultaneously the trnLF and ITS sequences or by cloning GI sequences. The species-specific markers reported in the present study have been tested in *Brachypodium* samples distributed predominantly in the Iberian Peninsula and with few samples from the Mediterranean region, and need to be confirmed in populations from other diverse origins.

### Genetics relationships and origin of Al-tolerant *B. hybridum* populations

Recently, Manzaneda *et al.* (2015) have shown that under water limitation allotetraploids maintain higher photosynthesis and stomatal conductance and show earlier flowering than diploids, concordant with a drought-escape strategy to cope with water stress. In our case, the ploidy level seems also to be important because the frequency of Al-tolerance is higher in allotetraploid than in diploid accessions (Contreras *et al.* 2014; results reported here). However, this is not the only factor explaining the Al-tolerance of *B. hybridum* because the majority of the allotetraploid plants are not tolerant. López-Álvarez *et al.* (2015) have found that *B. distachyon* grows in higher, cooler and wetter places; *B. stacei* in lower, warmer and drier places; and *B. hybridum* in places with intermediate climatic features than its diploid parental species. *Brachypodium hybridum* had the largest niche overlap with its parent niches, but a similar distribution range and niche breadth. López-Álvarez *et al.* (2015) also pointed out that ‘no evidence of niche divergence was found, suggesting that species diversification was not driven by ecological speciation but by evolutionary history, though it could be associated to distinct environmental adaptations’. Marques *et al.* (2017) have found that *B. distachyon* populations growing on basic soils were significantly more diverse than those growing in acidic soils. So, other important questions to be investigated are if the tolerant allotetraploid accessions have a common origin (genetic and/or geographic) and if they came from acidic soils and are adapted or not adapted to acidic conditions.

The genetic relationships inferred by SSR and ISSR neutral markers using different genetic estimators and clustering methods were always very similar, showing the allotetraploid-tolerant samples of *B. hybridum* grouped together in a separate cluster (Figs 3 and 4). This finding supports that tolerant allotetraploids have a common origin. This possibility is also supported by the presence of both exclusive SSR alleles and ISSR amplification products in the tolerant samples. Moreover, in the dendrogram obtained using ISSR and parsimony methods, and also in two dendrograms obtained with SSR markers, the Italian ABR8 tolerant diploid accession of *B. distachyon* is separated from the other sensitive *B. distachyon* accessions and falls near the allotetraploid-tolerant samples, though it is not grouped in the same cluster than *B. hybridum* Al-tolerant samples. The PCA developed with ISSR and SSR markers supported the genetic relationships previously obtained since it clearly separated the diploid (*B. distachyon*) and allotetraploid (*B. hybridum*) plants as well as the sensitive and tolerant accessions within each group (Fig. 4).

The *B. hybridum*-tolerant samples could came from the *B. distachyon*-tolerant accessions because all tolerant accessions (diploid and allotetraploids) have one insertion in the same position in the promoter region of *BdALMT1* gene (Contreras *et al.* 2014) and also they share common SSR and ISSR markers. By contrast, the nine *B. stacei* samples studied did not showed this insertion in the promoter region of *BdALMT1* gene. The insertion in the same position of the promoter in two different species and at different times is an event highly infrequent. The probability of association between this insertion and the Al-tolerance, if both were independent events (by chance), was estimated as 2.87 × 10^−9^ in allotetraploid plants (being significant at 5 %) (Contreras *et al.* 2014). Therefore, the co-occurrence of both events (insertion and tolerance) is not explained by chance. According to this, the *B. hybridum*-sensitive plants could come from diploid-sensitive accessions without this insertion, whereas the allotetraploid-tolerant plants could come from diploid-tolerant accessions. At present, we have only found one tolerant diploid accession (ABR8). More *B. distachyon* Al-tolerant accessions need to be characterized in order to have enough information to support this hypothesis. The nine *B. stacei* samples studied do not have the insertion in the promoter of the *BdALMT1* gene detected in all Al-tolerant accessions. However, the possibility that *B. stacei* Al-tolerant accessions not yet detected could be the parents of the *B. hybridum* Al-tolerant accessions cannot be excluded.

The lack of significant correlation between pairwise geographical and genetic distance matrices among *B. distachyon* accessions indicates there is no isolation-by-distance (IBD) within the studied samples. No significant correlation between pairwise geographical and genetic distance matrices was found among *B. hybridum* accessions yet. However, some genetic groups of samples could have the same geographical origin, as evidenced for the *B. hybridum* Al-tolerant accessions. In addition, *B. distachyon* samples from Iraq and Turkey are usually grouped together in dendrograms obtained with SSR and ISSR markers. By contrast, Marques *et al.* (2017) have found using a partial Mantel test a statistically significant IBD among the studied Iberian populations of *B. distachyon* as well as a statistically significant isolation-by-environment revealing the presence of environmental-driven isolation as one explanation for the genetic patterns found in the Iberian Peninsula for the diploid *B. distachyon* (Fig. 3). In addition, all the allotetraploid-tolerant accessions of *B. hybridum* came from western areas of the Iberian Peninsula (regions with acidic soils), whereas most the allotetraploid-sensitive accessions came from the eastern areas of the Iberian Peninsula (regions with non-acidic soils). It is important to remark that the only diploid-tolerant accession (ABR8) came from Siena, a region of Italy with acidic soils. This situation has also been observed in wheat and other cereals crops. So, several Al-tolerant wheat cultivars came from regions with acidic soils, such as Brazil and Portugal (Delhaize *et al.* 2012a; Garcia-Oliveira *et al.* 2013, 2014). Moreover, several tolerant barley cultivars from regions with acidic soils from China, Japan and Korea have been characterized and they have an insertion in the upstream region of *HvMATE1* Al-tolerance gene (Fujii *et al.* 2012). In addition, tolerant maize cultivars that have three copies of *ZmMATE1* Al-tolerance gene also have a common geographic origin in regions with acidic soils from South America (Maron *et al.* 2013). All these results suggest that Al-tolerance could be related with the adaptation to acidic soils. Ryan *et al.* (2010) have indeed suggested that the mechanisms of the gene expression regulation of Al-tolerance genes have been developed as a consequence of adaptation to acidic soils in a brief period of time. In these previous studies, genetic relationships were based on different candidate genes for Al-tolerance (mainly *ALMT* and *MATE*) that are probably under selection pressure. We present the first evidence of a common origin of Al-tolerance in the studied Iberian *B. hybridum* accessions using neutral molecular markers.

The common origin of the allotetraploid-tolerant accessions from the western areas of the Iberian Peninsula is supported by the results reported here for SSR and ISSR neutral molecular markers and also for the insertion in the promoter of the *BdALMT1* gene observed only in Al-tolerant accessions. One possible explanation that agrees with previous data obtained within populations of *B. distachyon* (Marques *et al.* 2017) is that the adaptation to acidic Al-rich soils caused isolation of these *B. hybridum* populations from the others.

## Conclusions

The genetic relationships obtained using SSR and ISSR neutral molecular markers support molecular evidence for the common origin of the Al-tolerant allotetraploid accessions from the western areas of the Iberian Peninsula.

An easy DNA barcoding method to discriminate among 52 *B. distachyon* samples, nine *B. stacei* samples and 33 *B. hybridum* samples with a 100 % of identification success (71 samples from Spain, one from Algeria, two from Portugal, one from Italy, one from France, one from Croatia, one from Ukraine, one from Iran, 10 from Turkey and five from Iraq) has been developed by amplifying the upstream-exon 1 region from the *BdALMT1* (*B. distachyon*) and *BsALMT1* (*B. stacei*) genes. Moreover, four SSR loci (Bd2GSSR_212, Bd4GSSR_399, Bd5GSSR_128 and Bd4GSSR_272) were obtained that can also distinguish among the three species within the studied samples.

## Conflicts of Interest

None declared.

## Sources of Funding

This work was supported by research grants AGL 2008-03049/AGR from the Ministerio de Educación y Ciencia of Spain and PR34/07-1581 from the Santander/Complutense.

## Contributions by the Authors

All authors have contributed to the manuscript and the research presented and they have participated in the discussion. R.C. and C.B. (ISSR markers, genetic relationships), R.C. and A.M.F. (SSR markers, genetic relationships), R.C., F.J.G., C.B. and E.B. (molecular markers to distinguish the three species of *Brachypodium*), R.C., A.J.M. and E.B. (collection of the samples).

## Supporting Information

The following additional information is available in the online version of this article—

**Table S1.** Diploid accessions of *Brachypodium distachyon* (2*n* = 2*x* = 10) and *B. stacei* (2*n* = 2*x* = 20), and allotetraploid accessions of *B. hybridum* (2*n* = 4*x* = 30) analysed.

**Table S2.** SSR markers analysed. Two different multiplex PCR with nine (Set 1) and eight (Set 2) different SSR markers were performed.

**Table S3.** PCR reagent concentration for the two multiplex SSR sets.

**Table S4.** Correlation coefficients (*r*) between pairwise geographical and genetic distance matrices based on SSRs.

**Table S5.** Private ISSR markers of *Brachypodium distachyon* and *B. hybridum* Al-tolerant samples.

**Figure S1.** Chromosomal location of the SSR markers designed from the nucleotide sequences of *Brachypodium distachyon* accession Bd21 chromosomes. The blue markers belong to the Multiplex Set 1 and the black markers belong to the Multiplex Set 2.

**Figure S2.** Molecular markers obtained by the amplification of the ALMT1 upstream-exon 1 region that distinguishes among the three species: *Brachypodium distachyon* (ABR1 755 accession), *B. hybridum* (CB accession) and *B. stacei* (E66 accession).

**Figure S3.** Dendrograms showing relationships of *Brachypodium distachyon* and *B. hybridum* accessions based on SSR markers using the Nei 72 and Cavalli-Sforza and Edwards distances, NJ grouping method and 1000 bootstrap replicates.

**Figure S4.** Dendrograms showing relationships of *Brachypodium distachyon* and *B. hybridum* accessions based on ISSR markers using SM, Dice and Jaccard coefficients, UPGMA grouping method and 1000 bootstrap replicates.

**Figure S5.** Principal component analysis (PCA) of *Brachypodium distachyon* and *B. hybridum* accessions based on ISSR data.

## Supplementary Material

Supporting-Information
