# Supplementary material for: Neutral molecular markers support common origin of aluminium tolerance in three congeneric grass species growing in acidic soils
Source: AoB Plants. 2017 Nov 7;9(6):plx060. doi: 10.1093/aobpla/plx060 (PMC5739048; doi:10.1093/aobpla/plx060)

**Supplementary Table S1.** Diploid lines of *B. distachyon* (2n=2x=10) and *B. stace*i (2n=2x= 20), and allotetraploid lines of *B. hybridum* (2n=4x=30) analysed

|  | Name | Nomenclature SSR and ISSR | Locality | Reference |
| --- | --- | --- | --- | --- |
| *B. distachyon* | Bd3-1 | Iraq 3 | Iraq | Garvin D. Lab |
|  | Bd21 | Iraq 4 | Iraq | Garvin D. Lab |
|  | ABR12 | Iraq 1 | Iraq | Jenkins *et al*. 2003 |
|  | ABR13 | Iraq 2 | Iraq | Jenkins *et al*. 2003 |
|  | Bd21-3 | Iraq 5 | Iraq | Vogel *et al*. 2008 |
|  | ABR1 | Turkey 1 | Kaman, Kiresehir, Turkey | Jenkins *et al*. 2003 |
|  | ABR11 | Turkey 2 | Manisa, Soma, Turkey | Jenkins *et al*. 2003 |
|  | ABR14 | Turkey 3 | Turkey | Jenkins *et al*. 2003 |
|  | BdTR2C | Turkey 9 | Kaşören, Turkey | Vogel *et al*. 2009 |
|  | BdTR12C | Turkey 8 | Beyvelioğlu, Turkey | Vogel *et al*. 2009 |
|  | BdTR13C | Turkey 6 | Afşar, Turkey | Vogel *et al*. 2009 |
|  | BdTR11H | Turkey 5 | Cambazdere, Turkey | Vogel *et al*. 2009 |
|  | BdTR5I | Turkey 4 | Yukarı Ç., Turkey | Vogel *et al*. 2009 |
|  | BdTR9K | Turkey 7 | Kireçköyü, Turkey | Vogel *et al*. 2009 |
|  | Adi 10 | Turkey 10 | Adiyaman, Turkey | Vogel *et al*. 2009 |
|  | Bd29-1 | Ukraine 1 | Ukraine | Garvin D Lab |
|  | ABR2 | France 1 | Octon, Herault, France | Jenkins *et al*. 2003 |
|  | ABR8 | Italy 1 | Siena, Italy | Jenkins *et al*. 2003 |
|  | ABR9 | Croatia 1 | Ljubljana, Croatia | Jenkins *et al*. 2003 |
|  | E4 | Albacete 1 | Albacete, Spain | Dr. E. Benavente |
|  | E7 | Badajoz 1 | Badajoz, Fregenal, Spain | Dr. E. Benavente |
|  | M23 | Cádiz 1 | Cádiz, Spain | Manzaneda *et al*. 2012 |
|  | M24 | Cádiz 2 | Cádiz, Spain | Manzaneda *et al*. 2012 |
|  | E10 | Cuenca 1 | Cuenca, Tórdiga, Spain | Dr. E. Benavente |
|  | E16 | Cuenca 2 | Cuenca, Spain | Dr. E. Benavente |
|  | E18 | Cuenca 3 | Cuenca, Río Cuervo, Spain | Dr. E. Benavente |
|  | E20 | Granada 1 | Granada, Onitar, Spain | Dr. E. Benavente |
|  | M21 | Granada 2 | Granada, Spain | Manzaneda *et al*. 2012 |
|  | E31 | Guadalajara 1 | Guadalajara, Spain | Dr. E. Benavente |
|  | E34 | Guadalajara 2 | Guadalajara, Spain | Dr. E. Benavente |
|  | E36 | Guadalajara 3 | Guadalajara, Spain | Dr. E. Benavente |
|  | E38 | Guadalajara 4 | Guadalajara, Spain | Dr. E. Benavente |
|  | ABR3 | Huesca 4 | Huesca, Ainsa, Spain | Jenkins *et al*. 2003 |
|  | E41 | Huesca 1 | Huesca, Spain | Dr. E. Benavente |
|  | E47 | Huesca 2 | Huesca, Escanilla, Spain | Dr. E. Benavente |
|  | E50 | Huesca 3 | Huesca, Nueno, Spain | Dr. E. Benavente |
|  | E58 | Jaén 3 | Jaén, El Tranco, Spain | Dr. E. Benavente |
|  | M30 | Jaén 4 | Jaén, Spain | Manzaneda *et al*. 2012 |
|  | E54 | Jaén 1 | Jaén, Paso Tiscar, Spain | Dr. E. Benavente |
|  | E57 | Jaén 2 | Jaén, Sierra del Segura, Spain | Dr. E. Benavente |
|  | M29 | Lerida 1 | Lérida, Spain | Manzaneda *et al*. 2012 |
|  | M32 | Murcia 1 | Murcia, Moratalla, Spain | Manzaneda *et al*. 2012 |
|  | ABR6 | Navarra 2 | Navarra, Arcos, Spain | Jenkins *et al*. 2003 |
|  | M22 | Navarra 1 | Navarra, Roncal, Spain | Manzaneda *et al*. 2012 |
|  | M31 | Palencia 1 | Palencia, Spain | Manzaneda *et al*. 2012 |
|  | E60 | Segovia 1 | Segovia, Spain | Dr. E. Benavente |
|  | E63 | Segovia 2 | Segovia, Carabias, Spain | Dr. E. Benavente |
|  | M26 | Valencia 1 | Valencia, Spain | Manzaneda *et al*. 2012 |
|  | M27 | Valencia 2 | Valencia, Spain | Manzaneda *et al*. 2012 |
|  | ABR7 | Valladolid 2 | Valladolid, Otero, Spain | Jenkins *et al*. 2003 |
|  | M25 | Valladolid 1 | Valladolid, Spain | Manzaneda *et al*. 2012 |
|  | E64 | Zaragoza 1 | Zaragoza, Frailes, Spain | Dr. E. Benavente |
|  |  |  |  |  |
|  | Name | Nomenclature SSR and ISSR | Locality | Reference |
| *B. stacei* | Oran |  | Oran, Algeria | Oran University |
|  | E66 |  | Almería, Cala Chica, Spain | Dr. E. Benavente |
|  | E67 |  | Almería, San José, Spain | Dr. E. Benavente |
|  | M17 |  | Almería, Sorbas, Spain | Manzaneda *et al*. 2012 |
|  | M18 |  | Almería, Cabo de Gata, Spain | Manzaneda *et al*. 2012 |
|  | M19 |  | Jaén, Tiscar, Spain | Manzaneda *et al*. 2012 |
|  | M20 |  | Jaén, Tiscar, Spain | Manzaneda *et al*. 2012 |
|  | E68 |  | Palmas, Lanzarote, Spain | Dr. E. Benavente |
|  | E69 |  | Tenerife, Gomera, Spain | Dr. E. Benavente |
| *B. hybridum* | GRA-788 | Iran 1 | Iran | IPK Gatersleben |
|  | M5 | Portugal 1 | Faro, Portugal | Manzaneda *et al*. 2012 |
|  | M7 | Portugal 2 | Mogadour, Portugal | Manzaneda *et al*. 2012 |
|  | M12 | Albacete 2 | Albacete, Nerio, Spain | Manzaneda *et al*. 2012 |
|  | E70 | Almería 3 | Almería, Spain | Dr. E. Benavente |
|  | E71 | Almería 1 | Almería, Cala, Spain | Dr. E. Benavente |
|  | E72 | Almería 2 | Almería, S. José, Spain | Dr. E. Benavente |
|  | Hoyo de Pinares | Ávila 1 | Ávila, Spain | Dr. M.A. Casado |
|  | Cabeza de Buey (CB) | Badajoz 3 | Badajoz, Spain | Dr. M.A. Casado |
|  | Castuera | Badajoz 2 | Badajoz, Spain | Dr. M.A. Casado |
|  | E73 | Badajoz 4 | Badajoz, Spain | Dr. E. Benavente |
|  | Serradilla | Cáceres 2 | Cáceres, Spain | Dr. M.A. Casado |
|  | M9 | Cáceres 1 | Cáceres, Monfragüe, Spain | Manzaneda *et al*. 2012 |
|  | M6 | Cadiz 3 | Cádiz, Algeciras, Spain | Manzaneda *et al*. 2012 |
|  | M3 | Ciudad Real 1 | Ciudad Real, Spain | Manzaneda *et al*. 2012 |
|  | M2 | Córdoba 1 | Córdoba, Spain | Manzaneda *et al*. 2012 |
|  | M8 | Gerona 1 | Gerona, Rosas M., Spain | Manzaneda *et al*. 2012 |
|  | M13 | Granada 3 | Granada, Cubillas, Spain | Manzaneda *et al*. 2012 |
|  | M16 | Granada 5 | Granada, Baza, Spain | Manzaneda *et al*. 2012 |
|  | E75 | Granada 4 | Granada, Spain | Dr. E. Benavente |
|  | Aracena | Huelva 3 | Huelva, Spain | Dr. M.A. Casado |
|  | Cortegana | Huelva 2 | Huelva, Spain | Dr. M.A. Casado |
|  | M15 | Huelva 1 | Huelva, Lepe, Spain | Manzaneda *et al*. 2012 |
|  | E76 | Huesca 5 | Huesca, Spain | Dr. E. Benavente |
|  | M1 | Jaén 6 | Jaén, Cazorla, Spain | Manzaneda *et al*. 2012 |
|  | E77 | Jaén 5 | Jaén, Spain | Dr. E. Benavente |
|  | E82 | Las Palmas 1 | Las Palmas, Betancu., Spain | Dr. E. Benavente |
|  | El Molar | Madrid 1 | Madrid, Spain | Dr. M.A. Casado |
|  | M14 | Murcia 2 | Murcia, Spain | Manzaneda *et al*. 2012 |
|  | M10 | Salamanca 1 | Salamanca, Río Águeda, Spain | Manzaneda *et al*. 2012 |
|  | E83 | Tenerife 1 | Tenerife, Buenavista, Spain | Dr. E. Benavente |
|  | E84 | Tenerife 2 | Tenerife, Gomera, Spain | Dr. E. Benavente |
|  | E81 | Zaragoza 2 | Zaragoza, Pto. Cavero, Spain | Dr. E. Benavente |

(*) The Populations/Lines underlined were classified as Al tolerant by relative root growth and different root histochemical staining methods. The Populations/Lines not underlined were classified as Al sensitive. The populations indicated with red color came from acidic soils.

- Jenkins G, Hasterok R, Draper J (2003) Building the molecular cytogenetic infrastructure of a new model grass. In Zwierzykowski Z, Surma M, Kachlicki P (eds) Application of Novel Cytogenetic and Molecular Techniques in Genetics and Breeding of the Grasses. Polish Academy of Sciences, Poznan, Poland, pp 77–84.
- Vogel JP, Garvin DF, Leong OM, Hayden DM (2006) Agrobacterium-mediated transformation and inbred line development in the model grass *Brachypodium distachyon*. Plant Cell Tissue Cult 84:199–211.
- Vogel JP, Tuna M, Budak H, Huo N, Gu YQ, Steinwand MA (2009) Development of SSR markers and analysis of diversity in Turkish populations *Brachypodium distachyon*. BMC Plant Biology 9:88.
- Vogel J, Hill T (2008) High-efficiency Agrobacterium-mediated transformation of *Brachypodium distachyon* inbred line Bd21-3. Plant Cell Rep 27:471-478.
- Garvin, D. Lab. USDA-ARS.
- IPK Gatersleben, Germany.
- Dr. M.A. Casado. Departamento de Ecología, Universidad Complutense de Madrid, Spain. Email: [mcasado@ucm.es](mailto:mcasado@ucm.es).

**Supplementary Table S2:** SSR markers analysed. Two different multiplex PCR with nine (Set 1) and eight (Set 2) different SSR markers were performed. The expected size and the chromosomal location were obtained from the sequence of Bd21 (The International *Brachypodium* Initiative, 2010). *The minimum number of repeat units showed here are the obtained in this study.

**Supplementary Table S3.** PCR reagent concentration for the two multiplex SSR Sets.

| Multiplex Set 1 | | | Multiplex Set 2 | | |
| --- | --- | --- | --- | --- | --- |
| 4.7 μL | DNA 23.5 ng | | 4.7 μL | DNA 23.5 ng | |
| 7 μL | Master Mix | | 7 μL | Master Mix | |
| 0 μL | H_2_O | | 0.5 μL | H_2_O | |
|  | Primer set | (μM) |  | Primer Set | (μM) |
|  | 1 | 0,127 |  | 10 | 0,064 |
|  | 2 | 0,127 |  | 11 | 0,064 |
|  | 3 | 0,127 |  | 12 | 0,075 |
|  | 4 | 0,127 |  | 13 | 0,075 |
| 2.3 μL | 5 | 0,127 | 1.8 μL | 14 | 0,128 |
|  | 6 | 0,149 |  | 15 | 0,128 |
|  | 7 | 0,149 |  | 16 | 0,214 |
|  | 8 | 0,149 |  | 17 | 0,214 |
|  | 9 | 0,149 |  |  |  |
| 14 μL | Total volume | | 14 μL | Total volume | |

**Supplementary Table S4a.** Correlation coefficients (r) between pairwise geographical distances and the pairwise distance matrices based on SSRs (Nei 72 and Cavalli-Sforza and Edwards distances) and ISSRs (SM, Dice and Jaccard coefficients) data. Independent Mantel test (Mantel 1967) for *B. distachyon* and *B. hybridum* lines were performed using the NTSYSpc 2.0 software. The number of permutations was 250 in all cases.

| Genetic matrices | *B. distachyon* | *B. hybridum* |
| --- | --- | --- |
| SSR markers and Nei 72 | 0.20973 | 0.13008 |
| SSR markers and Cavalli-Sforza and Edwards | 0.25607 | 0.12018 |
| ISSR markers and SM | 0.01531 | -0.12548 |
| ISSR markers and Dice | -0.01322 | -0.10015 |
| ISSR markers and Jaccard | -0.02850 | -0.10801 |

**Supplementary Table S4b.** Correlation coefficients (r) between different pairwise genetic matrices based on SSRs (Nei 72 and Cavalli-Sforza and Edwards distances) and ISSRs (SM, Dice and Jaccard coefficients) data. Independent Mantel test (Mantel 1967) for *B. distachyon* and *B. hybridum* lines were performed using the NTSYSpc 2.0 software. The number of permutations was 250 in all cases.

|  | *B. distachyon* | | | |  | *B. hybridum* | | | | |
| --- | --- | --- | --- | --- | --- | --- | --- | --- | --- | --- |
| Genetic matrices | Nei 72 | CaS-Ed | SM | Dice | Jaccard | Nei 72 | CaS-Ed | SM | Dice | Jaccard |
| Ne1 72 | 1 | 0.98216 | -0.37354 | -0.42442 | -0.42772 | 1 | 0.99031 | -0.86678 | -0.97193 | -0.85006 |
| CaS-Ed |  | 1 | -0.38545 | -0.43443 | -0.44206 |  | 1 | -0.86985 | -0.86818 | -0.84910 |
| SM |  |  | 1 | 0.96351 | 0.96326 |  |  | 1 | 0.97293 | 0.97355 |
| Dice | -0.02850 |  |  | 1 | 0.99134 |  |  |  | 1 | 0.99594 |
| Jaccard |  |  |  |  | 1 |  |  |  |  | 1 |

**Supplementary Table S5.** Private ISSR markers of *B. distachyon* and *B. hybridum* Al-tolerant samples.


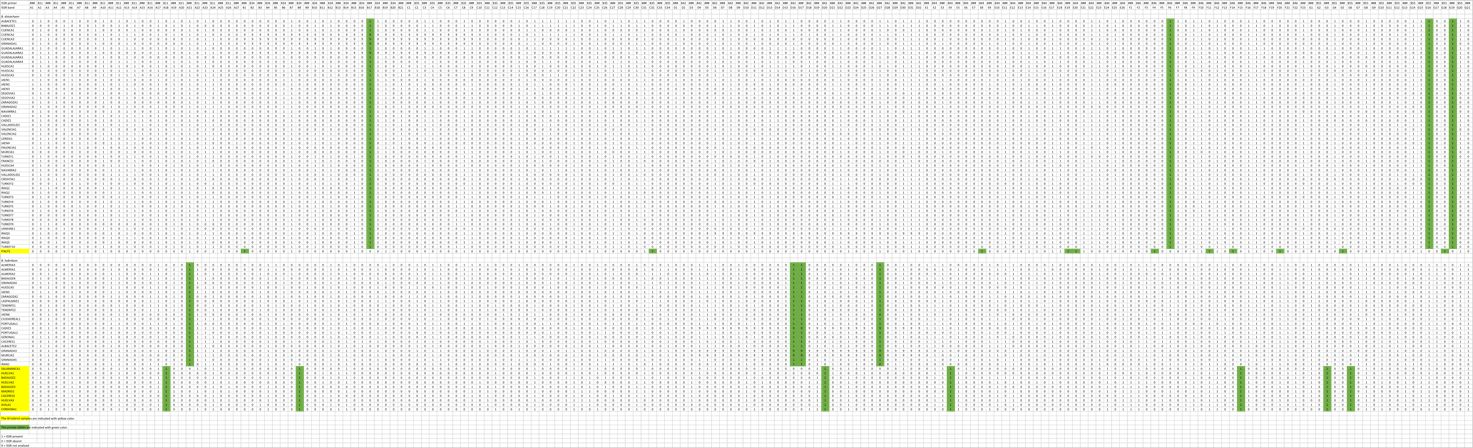


**Supplementary Figure S1.** Chromosomal location of the SSR markers designed from the nucleotide sequences of *B. distachyon* line Bd21 chromosomes. The blue markers belong to the Multiplex Set 1 and the black markers belong to the Multiplex Set 2.


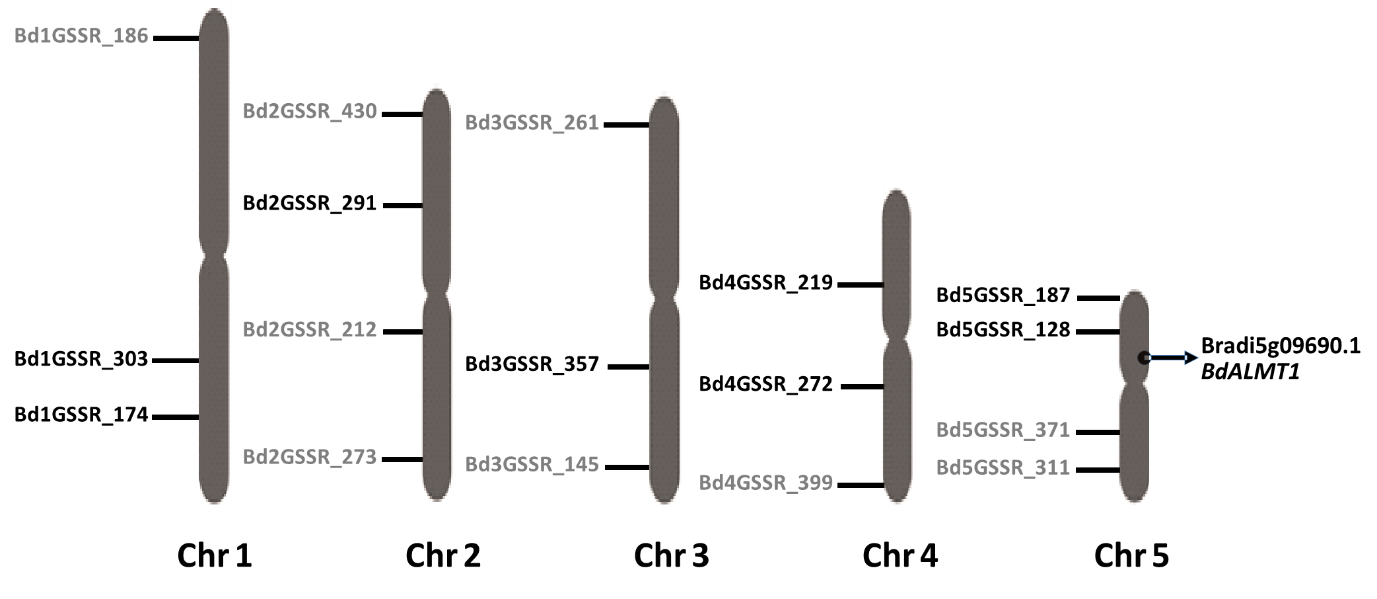


**Supplementary Figure S2:** (a). Molecular marker obtained by the amplification of the *ALMT1* upstream-exon 1 region that distinguishes among the three species: *B. distachyon* (ABR1 line), *B. hybridum* (CB line) and *B. stacei* (E66 line). (b). Differentiation by the Bd4GSSR_399 SSR marker of the three cytotypes. The amplified products were separated on 1.2% agarose gels. SM: size molecular marker (100 bp).

**Supplementary Figure S3**


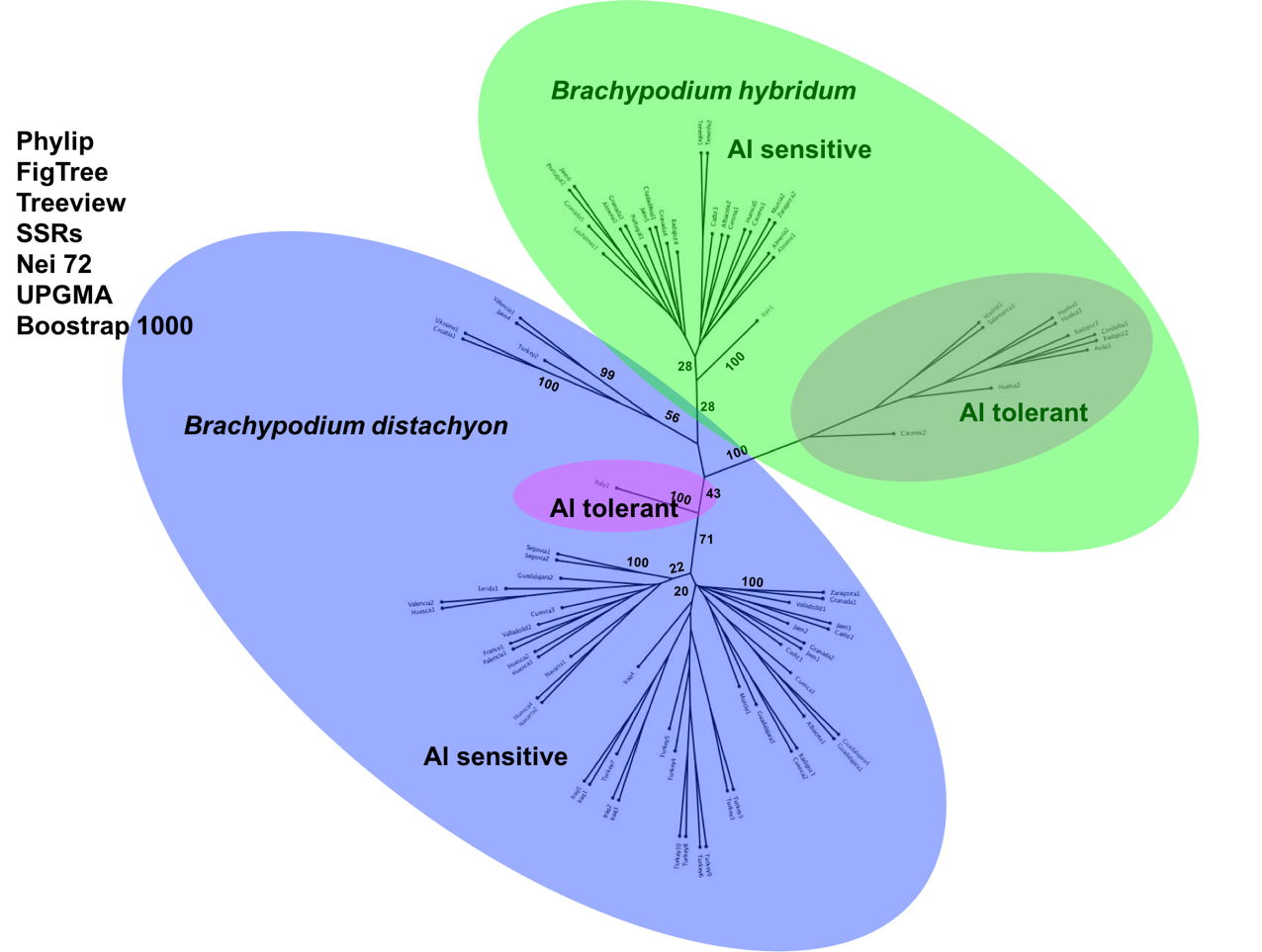


**Supplementary Figure S3**


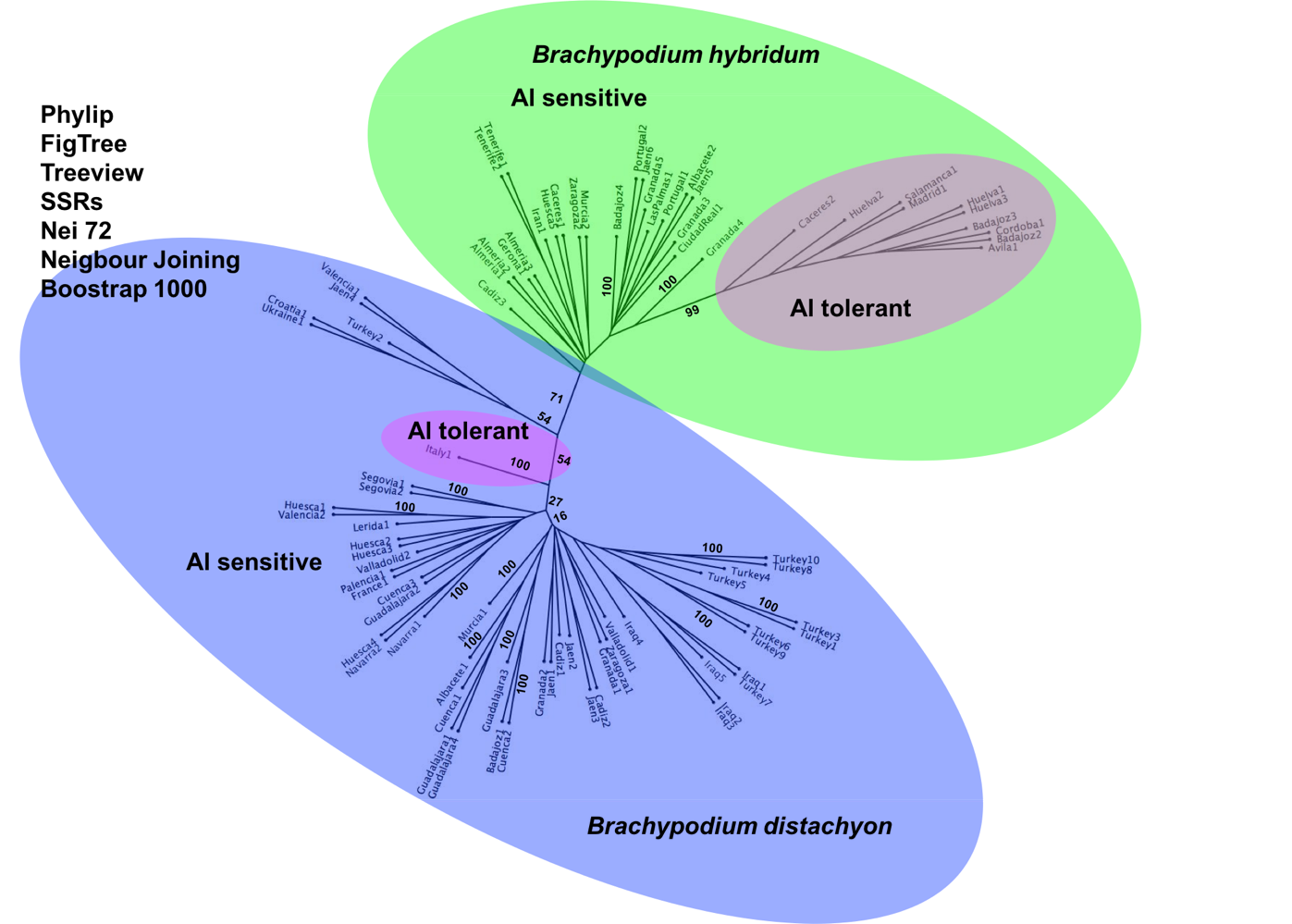


**Supplementary Figure S3**


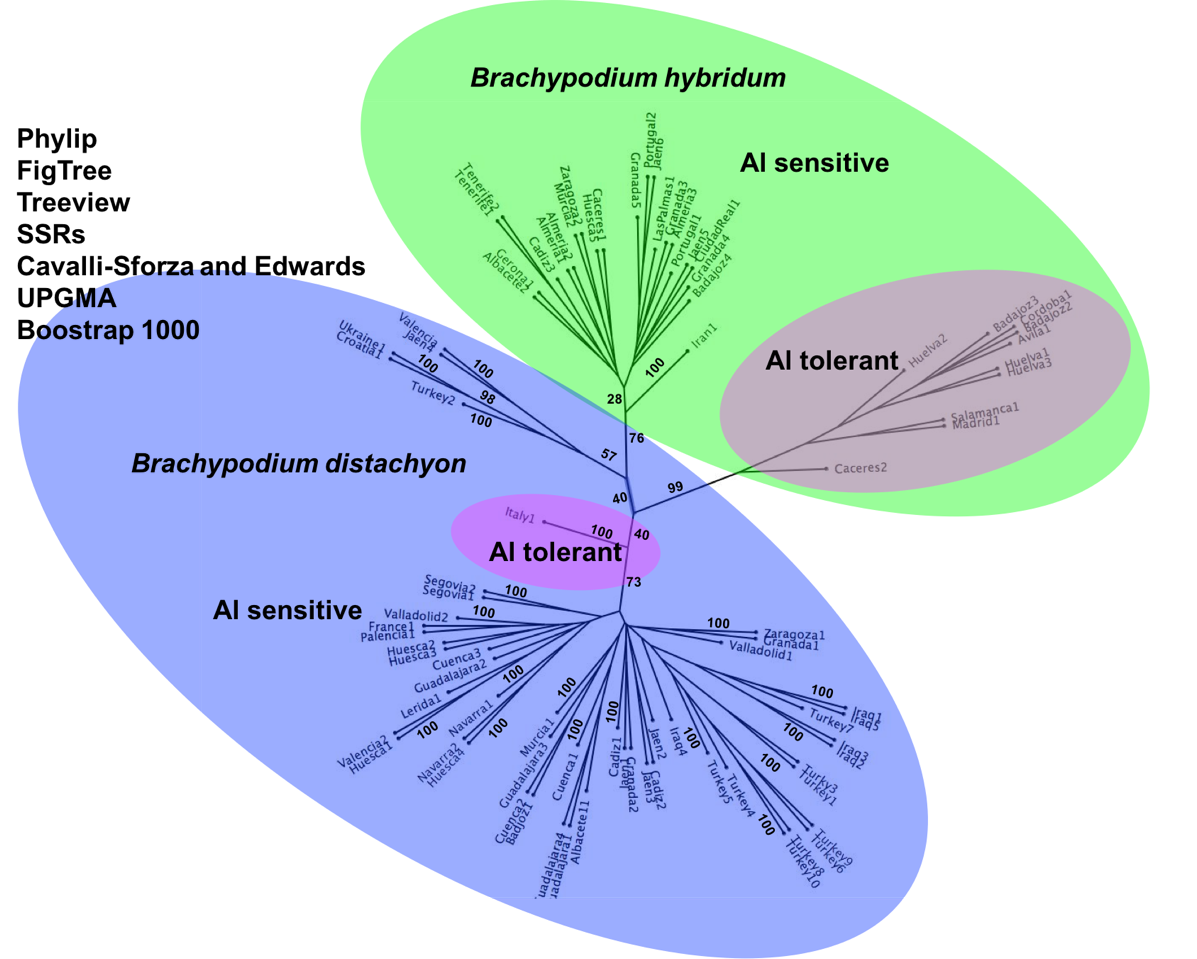


**Supplementary Figure S3**


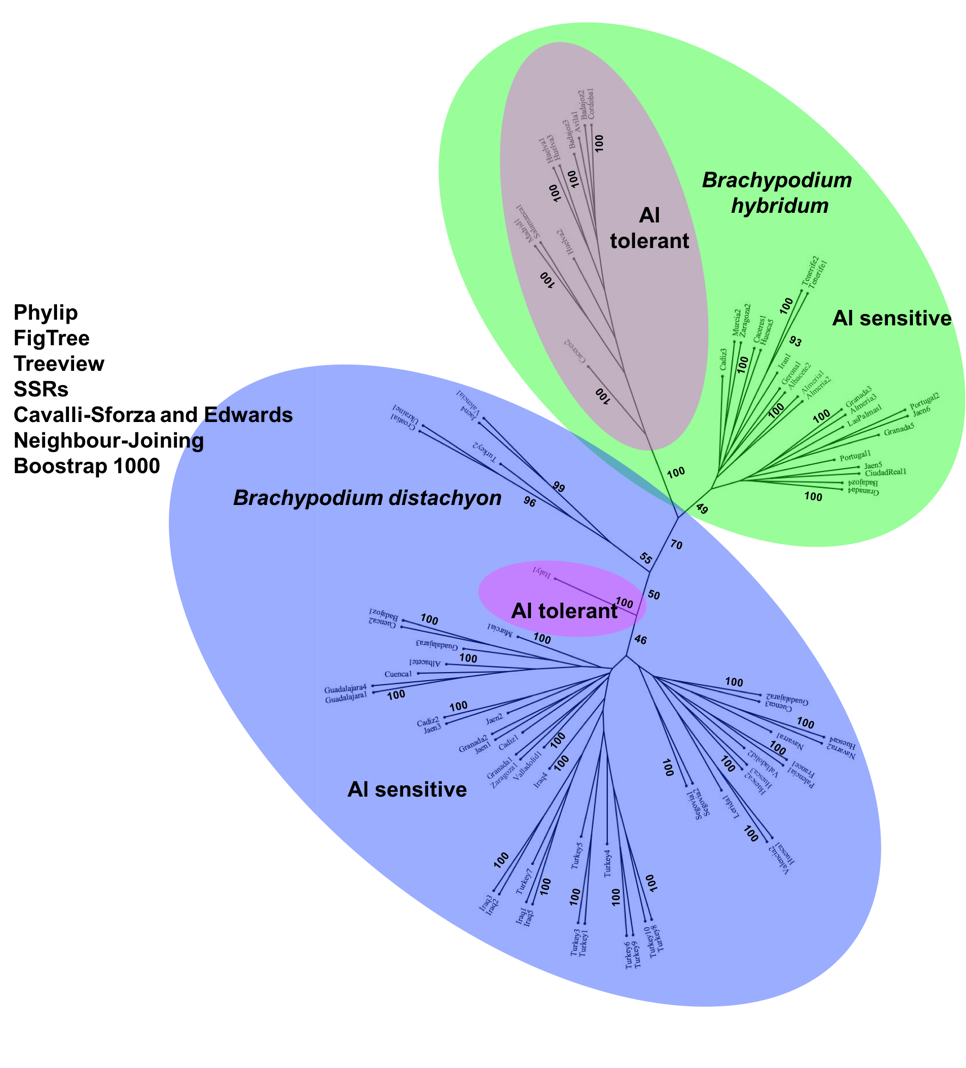


**Supplementary Figure S4**. Dendrograms obtained with *B. distachyon*, *B. hybridum* lines and with ISSR markers using SM, Dice and Jaccard coefficients, UPGMA grouping method and 1,000 bootstrap replicates.


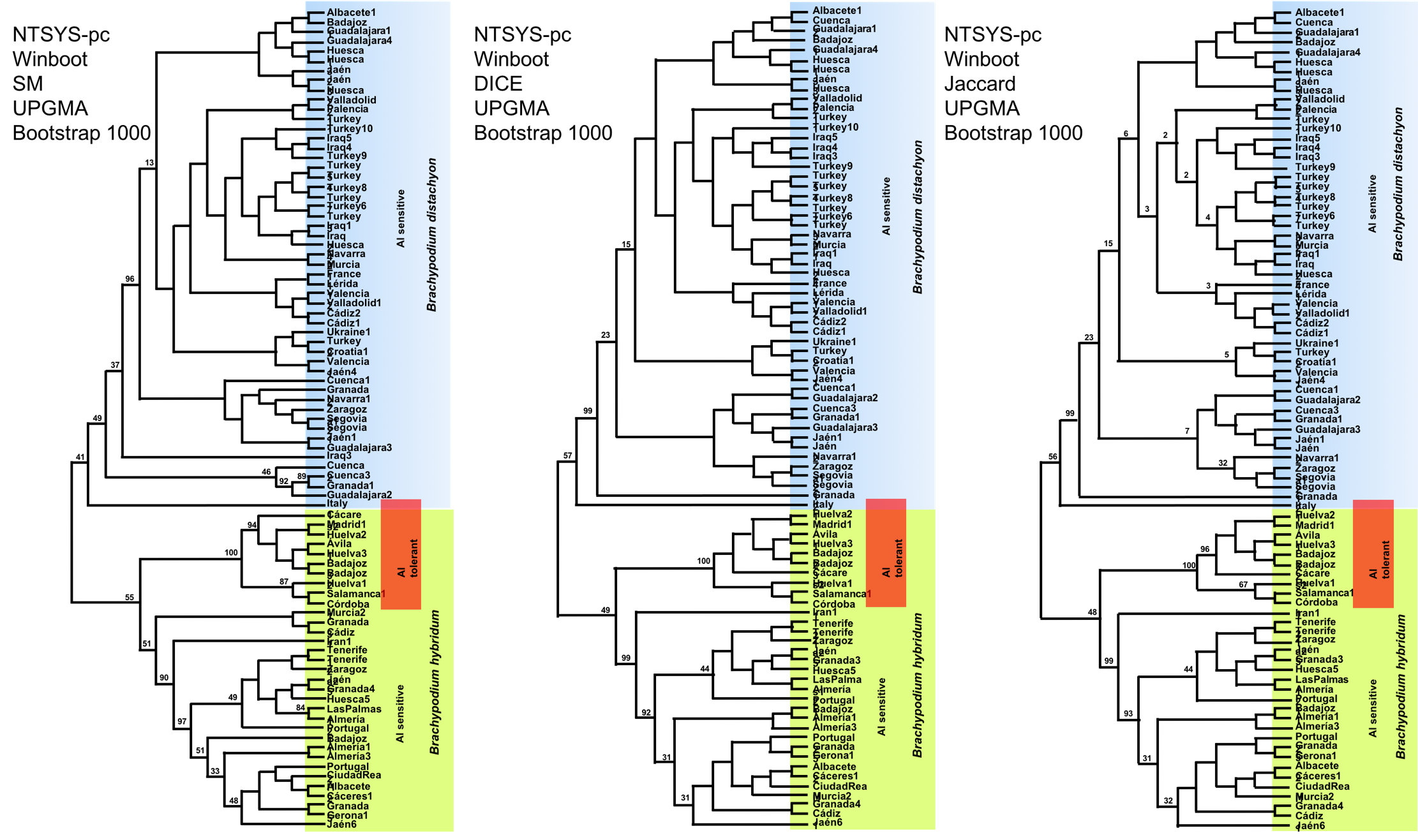


**Supplementary Figure S5**. Bidimensional plot of *B. distachyon* and *B. hybridum* accessions revealed by PCA analysis based on ISSR data using the Nei 72 genetic distance. The original name of the lines is indicated in Supplementary Table S1.


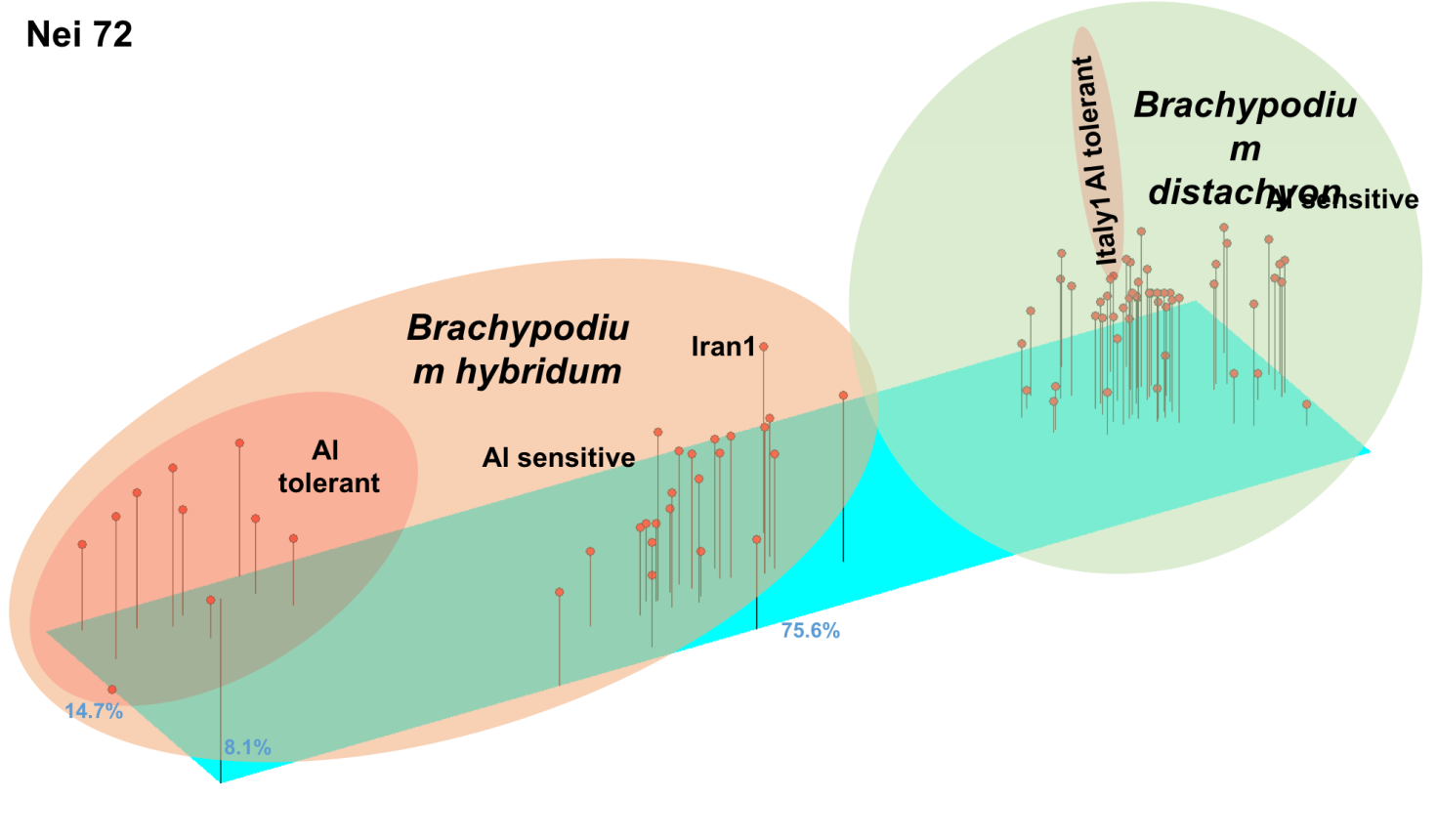


**Supplementary Figure S5**. Bidimensional plot of *B. distachyon* and *B. hybridum* accessions revealed by PCA analysis based on ISSR data using the SM coefficient. The original name of the lines is indicated in Supplementary Table S1.


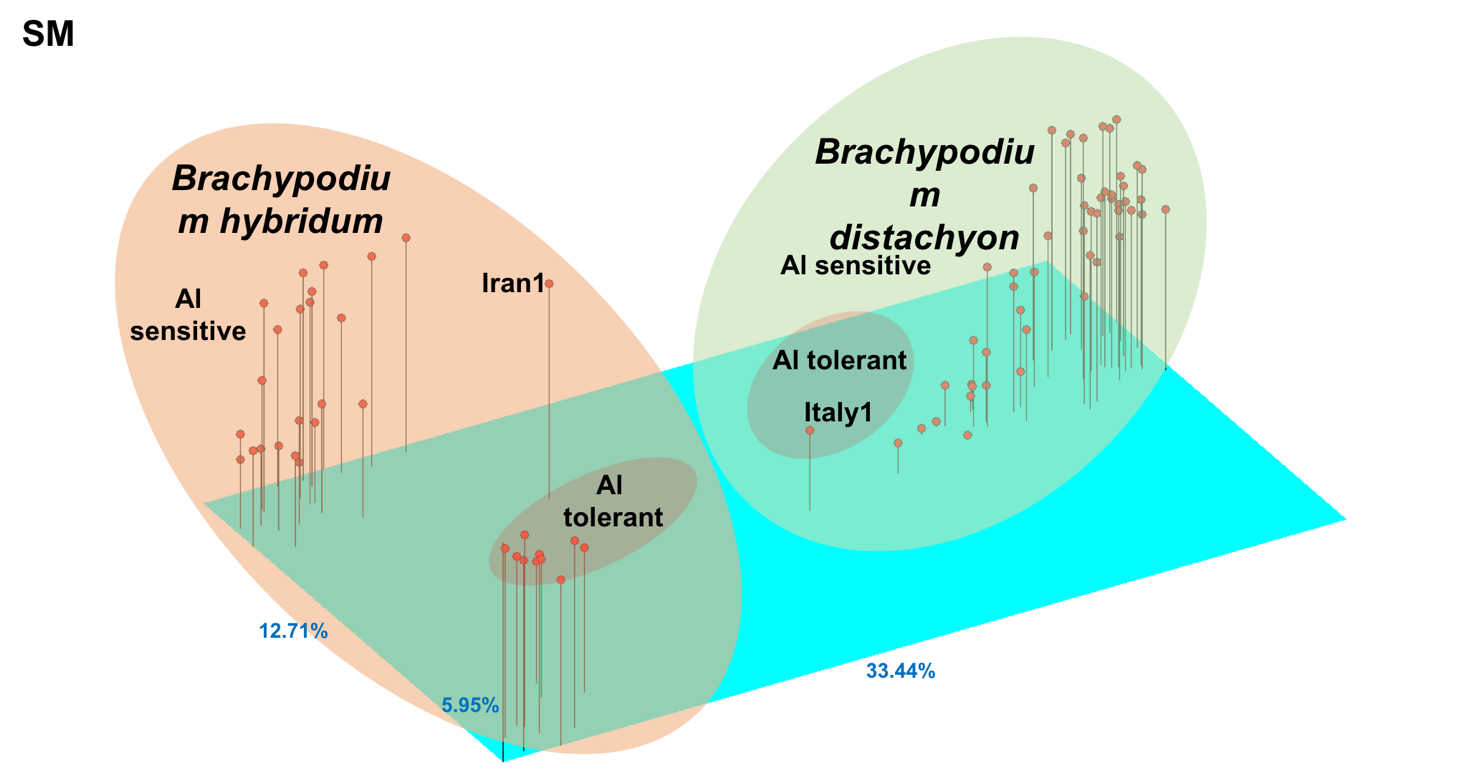


**Supplementary Figure S5**. Bidimensional plot of *B. distachyon* and *B. hybridum* accessions revealed by PCA analysis based on ISSR data using the Dice coefficient. The original name of the lines is indicated in Supplementary Table S1.


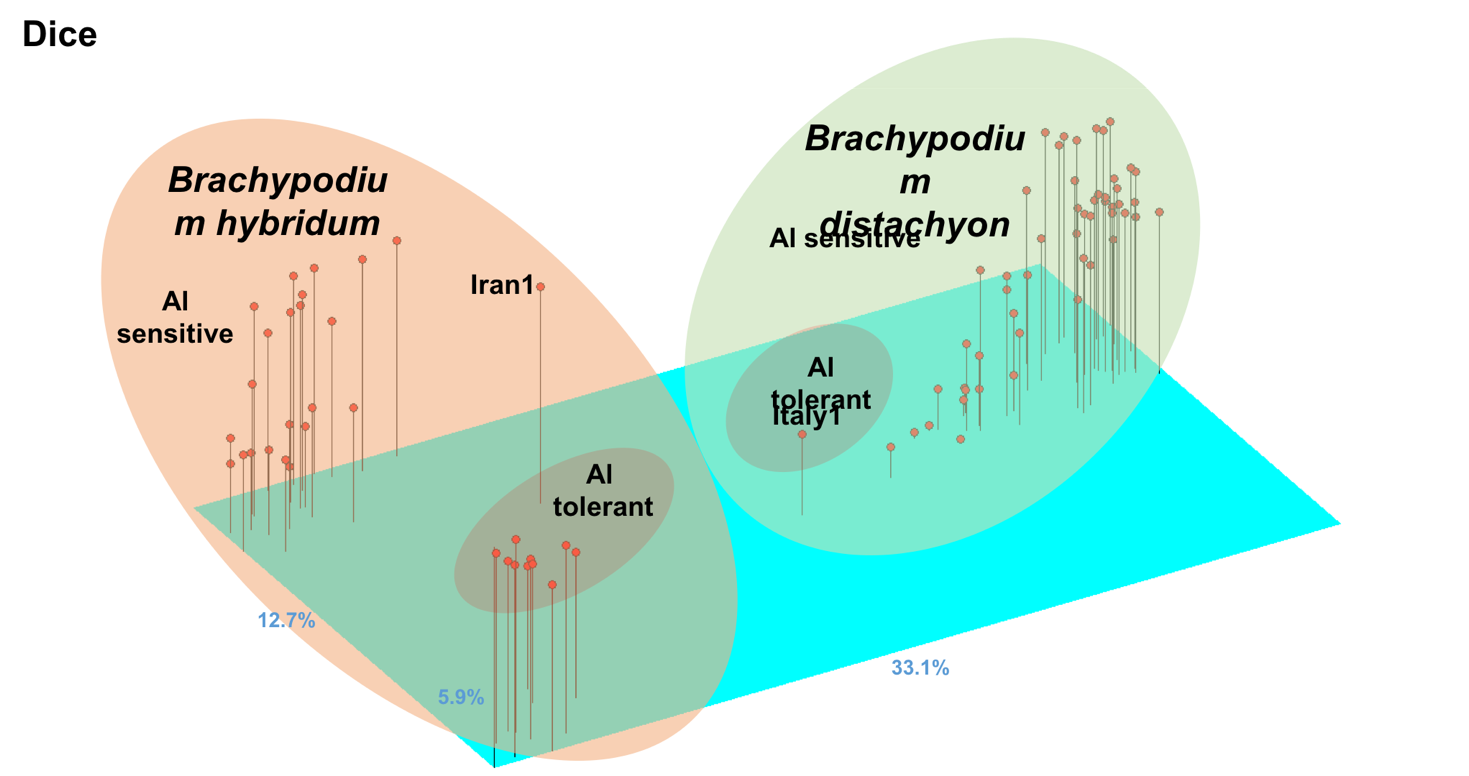


**Supplementary Figure S5**. Bidimensional plot of *B. distachyon* and *B. hybridum* accessions revealed by PCA analysis based on ISSR data using the Jaccard coefficient. The original name of the lines is indicated in Supplementary Table S1.


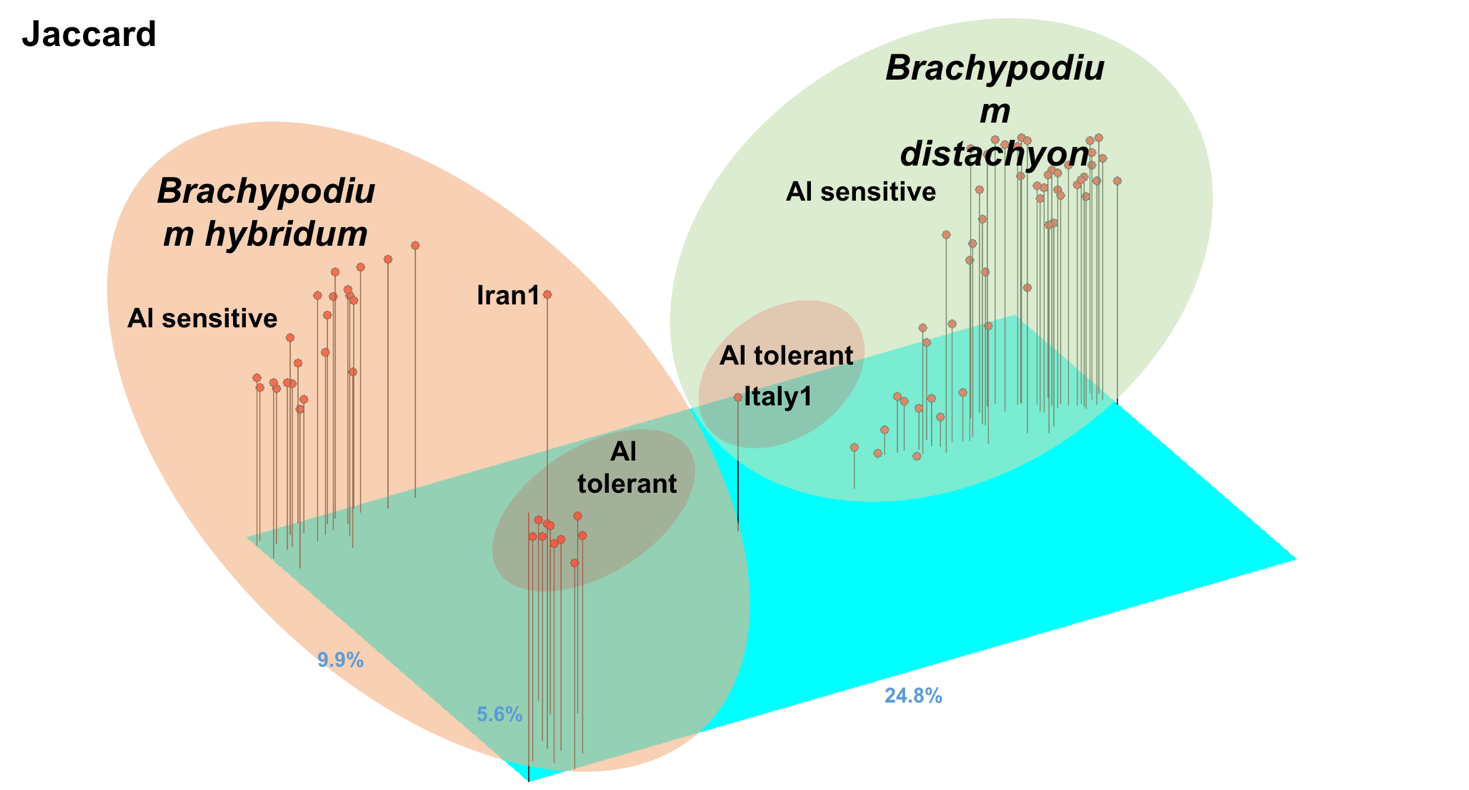


**Supplementary Figure S5**. Bidimensional plot of *B. distachyon* and *B. hybridum* accessions revealed by PCA analysis based on ISSR data using the SM coefficient. The original name of the lines is indicated in Supplementary Table S1.


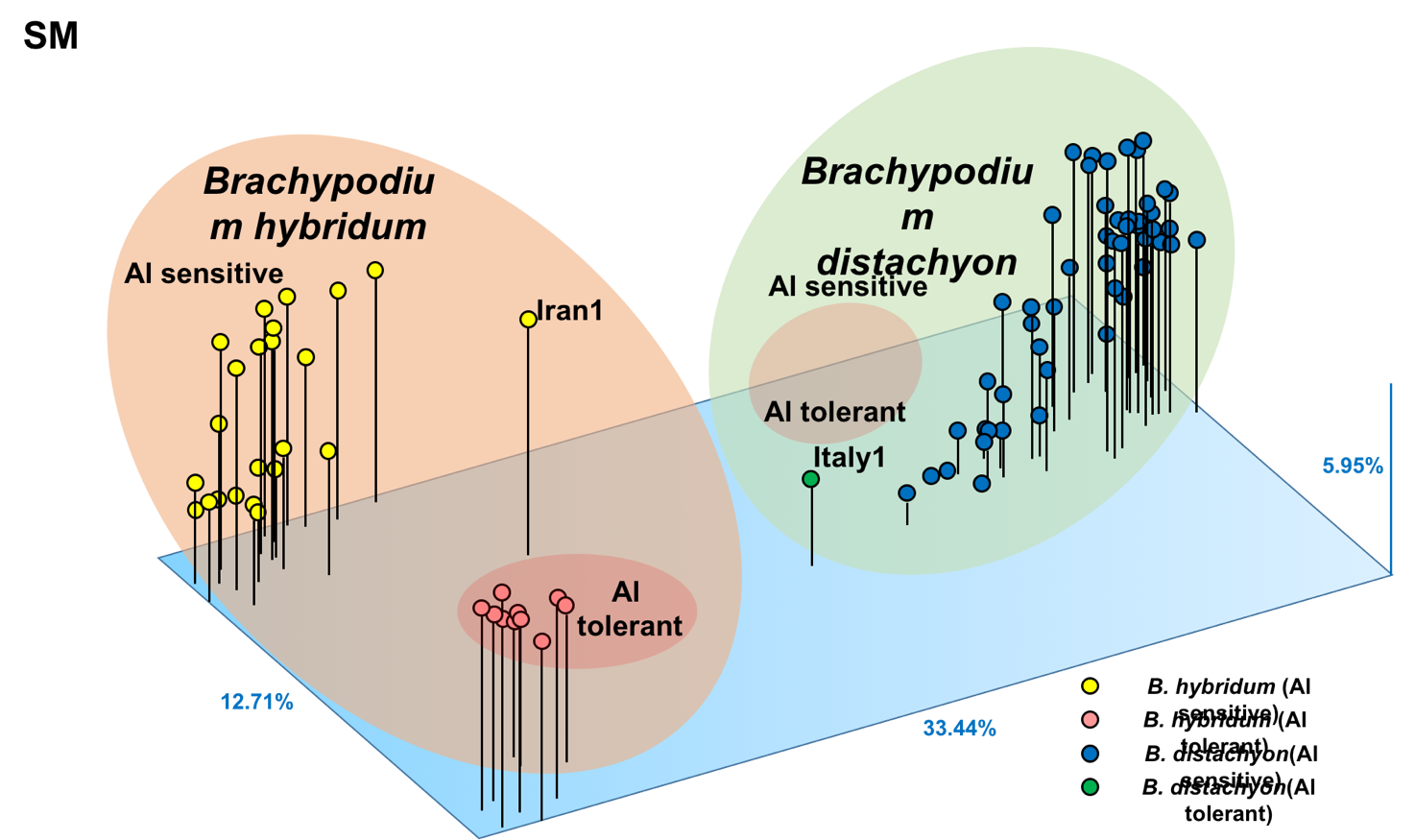

Supplement: Supporting-Information [file plx060_suppl_supporting-information.docx]
